# Supplementary material for: Selection and Characterization of Artificial Proteins Targeting the Tubulin α Subunit
Source: Structure. 2019 Mar 5;27(3):497–506.e4. doi: 10.1016/j.str.2018.12.001 (PMC6408325; doi:10.1016/j.str.2018.12.001)
Supplement: Document S1. Figures S1–S3 [file mmc1.pdf]

Structure, Volume *27*

Supplemental Information

Selection and Characterization of Artificial Proteins Targeting the Tubulin α Subunit

Valérie Campanacci, Agathe Urvoas, Tanja Consolati, Soraya Cantos-Fernandes, Magali Aumont-Nicaise, Marie Valerio-Lepiniec, Thomas Surrey, Philippe Minard, and Benoît Gigant

```
            S1 T1          H1                           H1'  S1'        S1"    S2 T2     H2
a-Tubulin  MRECISIHVGQAGVQIGNACWELYCLEHGIQPDGQMPSDKTIGGGDDSFNTFFSETGAGKHVPRAVFVDLEPTVIDEVRT
                    10         20         30         40         50         60         70         80
b-Tubulin  MREIVHIQAGQCGNQIGAKFWEVISDEHGIDPTGSYHGDSDLQL..ERINVYYNEATGNKYVPRAILVDLEPGTMDSVRS
            S1 T1          H1                     H1'  H1'  S1'        S1"    S2 T2     H2

          H2'  H2" S3    T3      H3'         H3                   S4    T4        H4
a-Tubulin  GTYRQLFHPEQLITGKEDAANNYARGHYTIGKEIIDLVLDRIRKLADQCTGLQGFLVFHSFGGGTGSGFTSLLMERLSVD
                    90        100        110        120        130        140        150        160
b-Tubulin  GPFGQIFRPDNFVFGQSGAGNNWAKGHYTEGAELVDSVLDVVRKESESCDCLQGFQLTHSLGGGTGSGMGTLLISKIREE
          H2'  H2" S3    T3                H3                     S4    T4       H4

              S5      T5            H5          S6 T6     H6                     H7
a-Tubulin  YGKKSKLEFSIYPAPQVSTAVVEPYNSILTTHTTLEHSDCAFMVDNEAIYDICRRNLDIERPTYTNLNRLISQIVSSITA
                   170        180        190        200        210        220        230        240
b-Tubulin  YPDRIMNTFSVMPSPKVSDTVVEPYNATLSVHQLVENTDETYCIDNEALYDICFRTLKLTTPTYGDLNHLVSATMSGVTT
              S5      T5            H5          S6 T6     H6                     H7

 H7    T7      H8             S7      M loop          H9      H9'      H9"     S8
a-Tubulin  SLRFDGALNVDLTEFQTNLVPYPRIHFPLATYAPVISAEKAYHEQLSVAEITNACFEPANQMVKCDPRHGKYMACCLLYR
                   250        260        270        280        290        300        310        320
b-Tubulin  CLRFPGQLNADLRKLAVNMVPFPRLHFFMPGFAPLTSRGSQQYRALTVPELTQQMFDSKNMMAACDPRHGRYLTVAAIFR
 H7    T7      H8             S7      M loop          H9      H9'      H9"     S8

                H10                   S9                      S10             H11
a-Tubulin  GDVVPKDVNAAIATIKTKRSIQFVDWCPTGFKVGINYQPPTVVPGGDLAKVQRAVCMLSNTTAIAEAWARLDHKFDLMYA
                   330        340        350        360        370        380        390        400
b-Tubulin  GRMSMKEVDEQMLNVQNKNSSYFVEWIPNNVKTAVCDIPP--------RGLKMSATFIGNSTAIQELFKRISEQFTAMFR
                H10                   S9                      S10             H11

             H11'            H12
a-Tubulin  KRAFVHWYVGEGMEEGEFSEAREDMAALEKDYEEVGVDSVEGEGEEEGEEY
                   410        420        430        440        450
b-Tubulin  RKAFLHWYTGEGMDEMEFTEAESNMNDLVSEYQQYQDATADEQGEFEEEEGEDEA
             H11'            H12
```

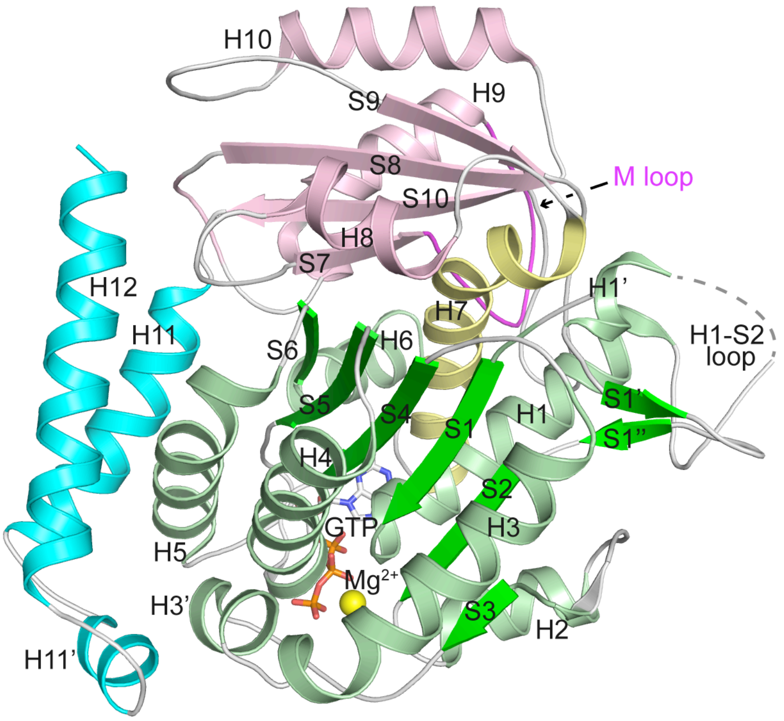

Figure S1. Sequences of α- and β-tubulin, secondary structure assignments and domain definition, Related to Figures 2, 3 and 5. (Top) The sequences of the *Bos taurus* α1B and β2B tubulin isotypes have been aligned as in (Löwe et al., 2001). These sequences were used to refine the tubulin–iE5 and tubulin–iiH5 structures because, to the best of our knowledge, those of *Ovis aries* tubulin are not known. The secondary

structure nomenclature is as in (Löwe et al., 2001) and the boundaries of the helices (highlighted in cyan) and strands (yellow) were determined with Pymol from the following structures: pdb id 6GWC (this work), 5EYP (Ahmad et al., 2016), and 4I4T (Prota et al., 2013a), which comprises a helical motif in the M loop as shown here. (Bottom) Tubulin domains. The α subunit (pdb id 5EYP) is shown. The secondary structure elements of its N-terminal domain are in green, with the strands in brighter color; those of the intermediate domain are in pink; and the helices of the C-terminal domain are in cyan. The H7 helix is in yellow and the M loop in magenta.

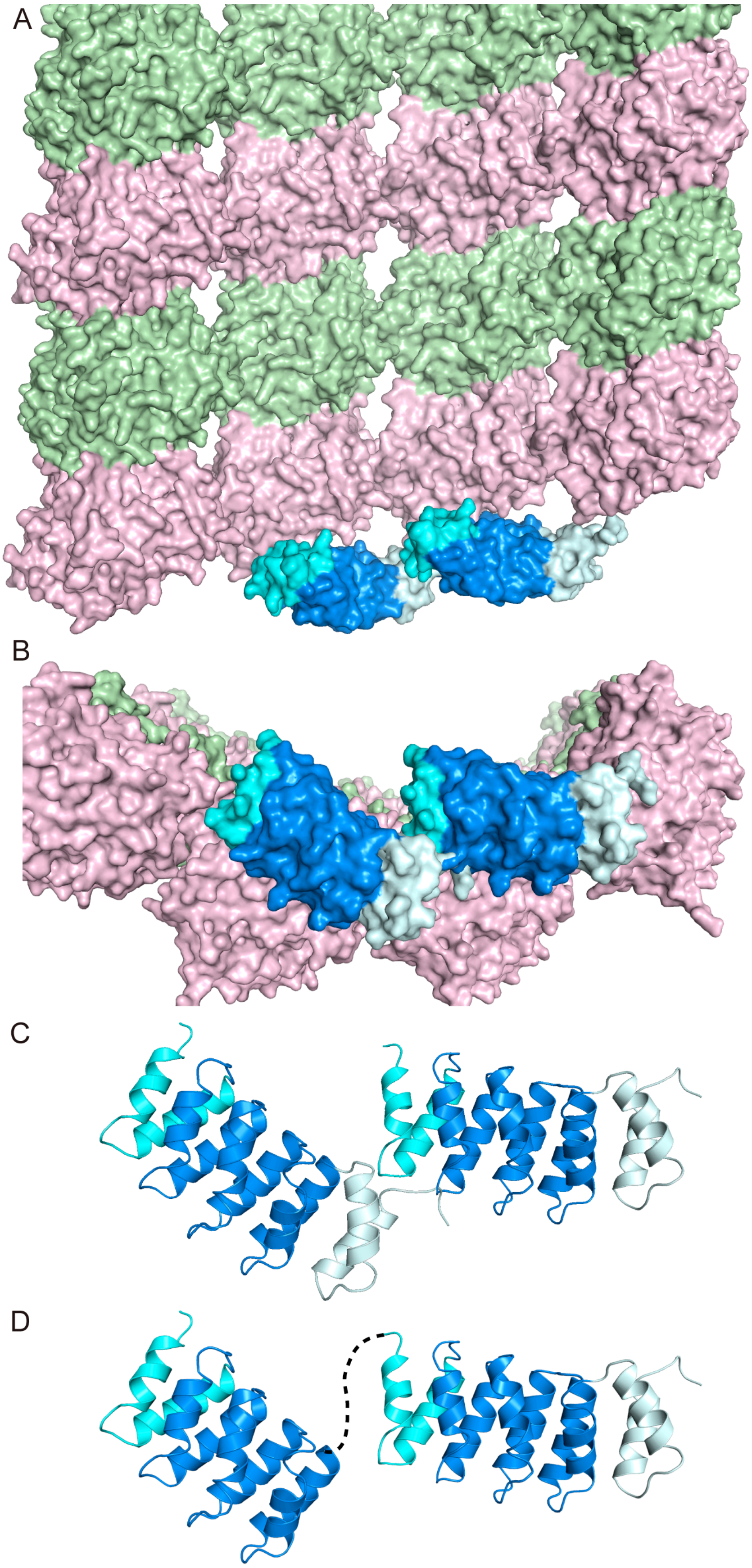

Figure S2. Design of the (iiH5)$_2$ tandem repeat αRep, Related to Figures 1H, 3 and 4. (A,B) Model of iiH5 bound at the (-) end of a microtubule. This model was obtained by superposing α-tubulin in tubulin–iiH5 to α-tubulin in the microtubule structure (pdb id 3JAK). Four protofilaments are drawn, two of them

being decorated by a iiH5 molecule. Views from the inside of the microtubule (A) and along the microtubule axis (B). Color code as in Fig. 3A except that the C-cap of iiH5 is in lighter cyan. (C) Same orientation as in panel B, but only the two iiH5 molecules are shown. (D) Design of $(iiH5)_2$. To build the tandem repeat αRep, the C-cap of one αRep was removed and the C-terminal end of its last internal HEAT repeat was linked to the N-terminal end of the N-cap of the second one using a $(GGGGS)_3$-GGS motif (dashed line).

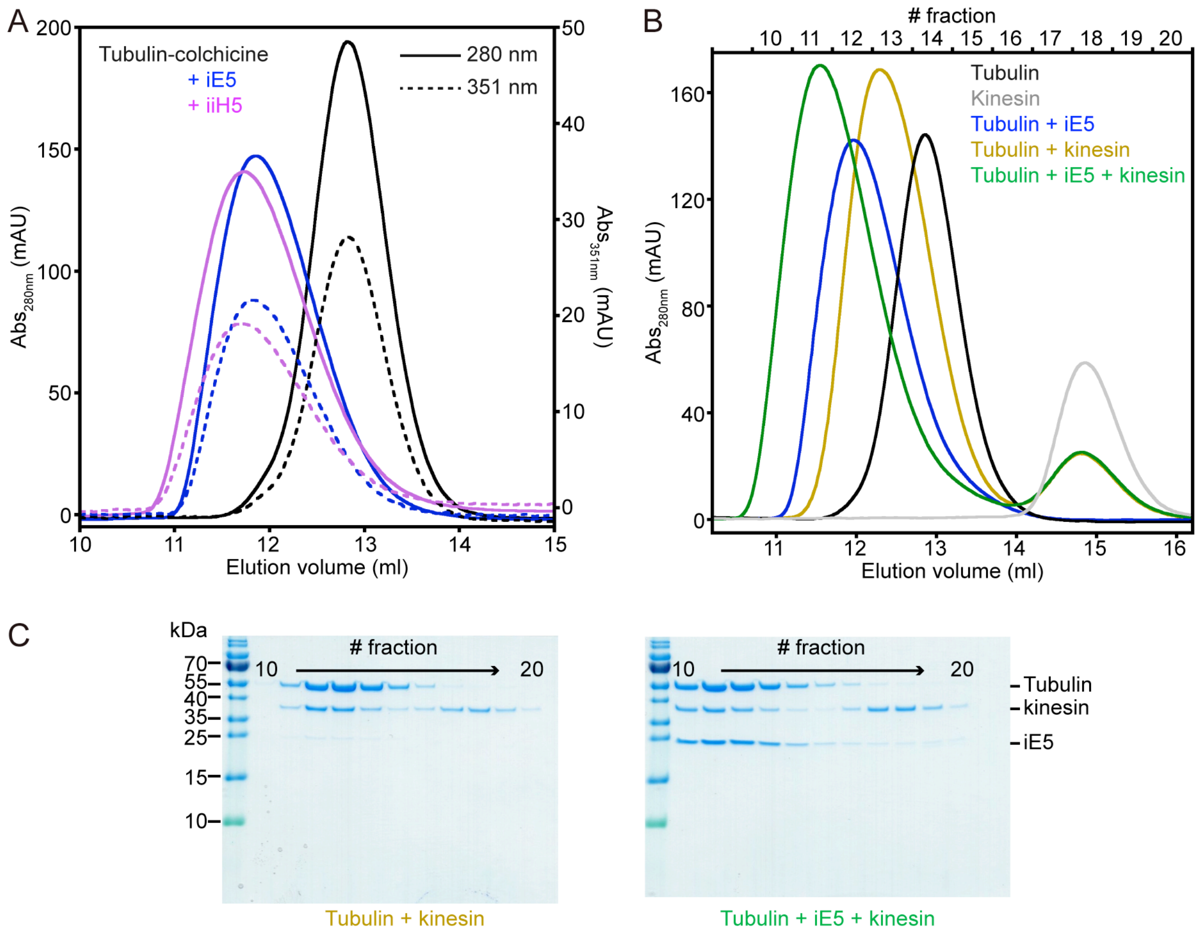

Figure S3. iE5 interacts with tubulin bound to colchicine or to kinesin-1, Related to Figures 1B and 5. (A) iE5 and iiH5 interact with the tubulin–colchicine complex. Gel filtration profile in 20 mM Pipes-K, pH 6.8, 1 mM $MgCl_2$, and 0.5 mM EGTA, of 20 μM tubulin–colchicine alone (black lines) or with 80 μM iE5 (blue) or iiH5 (magenta). The absorbance signal of colchicine at 351 nm is also shown (dashed lines). (B,C) iE5 makes a ternary complex with tubulin and kinesin-1. (B) Gel filtration profile in the buffer used in panel A of tubulin (20 μM), of kinesin-1 motor domain (30 μM), or of different mixtures of tubulin, kinesin, and iE5 (80 μM), as indicated. The main chromatographic peak of the tubulin:iE5:kinesin sample is shifted compared to those of tubulin:kinesin and tubulin:iE5, indicating the formation of the ternary complex. (C) Fractions defined at the top of panel B were submitted to SDS-PAGE in the case of tubulin:kinesin (Left) and tubulin:iE5:kinesin (Right), confirming the formation of a ternary complex in this last case.
