# Supplementary material for: Selection and Characterization of Artificial Proteins Targeting the Tubulin α Subunit
Source: Structure. 2019 Mar 5;27(3):497–506.e4. doi: 10.1016/j.str.2018.12.001 (PMC6408325; doi:10.1016/j.str.2018.12.001)
Supplement: Document S2. Article plus Supplemental Information [file mmc2.pdf]

Structure

Article

Selection and Characterization of Artificial Proteins Targeting the Tubulin α Subunit

Graphical Abstract

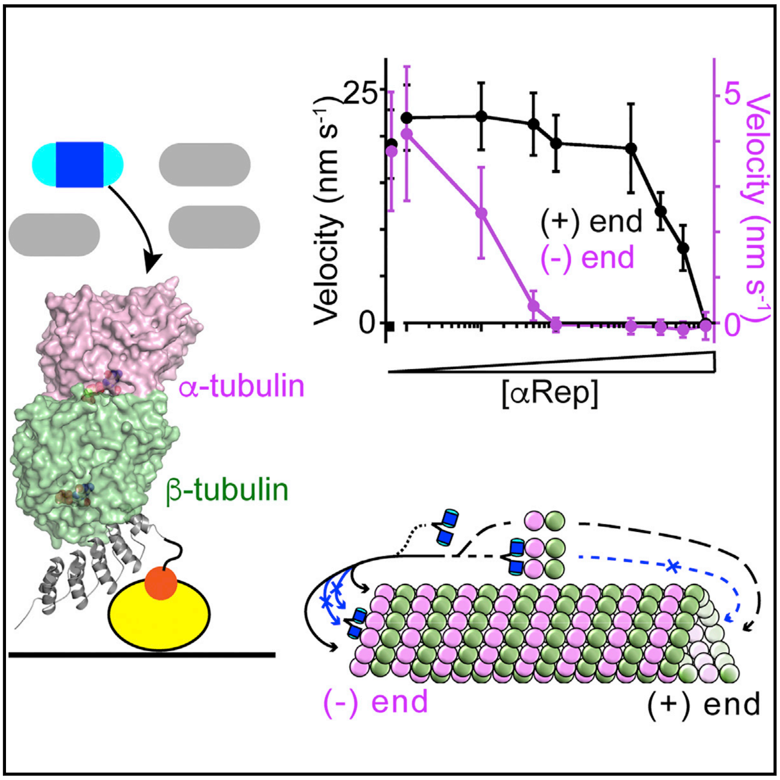

Highlights

- Selection of α-tubulin-specific artificial αRep proteins
- The αReps inhibit microtubule assembly and specifically block growth at the (–) end
- The αReps target the longitudinal surface of α-tubulin
- The αReps are useful tools for the mechanistic study of microtubule dynamics

Authors

Valérie Campanacci, Agathe Urvoas, Tanja Consolati, ..., Thomas Surrey, Philippe Minard, Benoît Gigant

Correspondence

philippe.minard@i2bc.paris-saclay.fr (P.M.),
benoit.gigant@i2bc.paris-saclay.fr (B.G.)

In Brief

Campanacci et al. have selected artificial proteins, termed αReps, that bind to the tubulin alpha subunit. These αReps inhibit microtubule assembly with a specific blocking effect at the slower growing microtubule minus end. The structure of tubulin-αRep complexes gives a rationale for this mechanism.

Campanacci et al., 2019, Structure *27*, 497–506
March 5, 2019 © 2018 The Author(s). Published by Elsevier Ltd.
https://doi.org/10.1016/j.str.2018.12.001

Cell**Press**

Selection and Characterization of Artificial Proteins Targeting the Tubulin α Subunit

Valérie Campanacci,[1,3] Agathe Urvoas,[1,3] Tanja Consolati,[2] Soraya Cantos-Fernandes,[1] Magali Aumont-Nicaise,[1] Marie Valerio-Lepiniec,[1] Thomas Surrey,[2] Philippe Minard,[1,*] and Benoît Gigant[1,4,*]
[1]Institute for Integrative Biology of the Cell (I2BC), CEA, CNRS, Univ. Paris-Sud, Université Paris-Saclay, Gif-sur-Yvette Cedex 91198, France
[2]The Francis Crick Institute, 1 Midland Road, London NW1 1AT, UK
[3]These authors contributed equally
[4]Lead Contact
*Correspondence: philippe.minard@i2bc.paris-saclay.fr (P.M.), benoit.gigant@i2bc.paris-saclay.fr (B.G.)
https://doi.org/10.1016/j.str.2018.12.001

SUMMARY

Microtubules are cytoskeletal filaments of eukaryotic cells made of αβ-tubulin heterodimers. Structural studies of non-microtubular tubulin rely mainly on molecules that prevent its self-assembly and are used as crystallization chaperones. Here we identified artificial proteins from an αRep library that are specific to α-tubulin. Turbidity experiments indicate that these αReps impede microtubule assembly in a dose-dependent manner and total internal reflection fluorescence microscopy further shows that they specifically block growth at the microtubule (−) end. Structural data indicate that they do so by targeting the α-tubulin longitudinal surface. Interestingly, in one of the complexes studied, the α subunit is in a conformation that is intermediate between the ones most commonly observed in X-ray structures of tubulin and those seen in the microtubule, emphasizing the plasticity of tubulin. These α-tubulin-specific αReps broaden the range of tools available for the mechanistic study of microtubule dynamics and its regulation.

INTRODUCTION

Microtubules are eukaryotic cytoskeletal assemblies involved in critical functions ranging from intracellular trafficking to ciliogenesis and cell division. To achieve these different functions, cells constantly reorganize their microtubule network, regulating microtubule nucleation and dynamics. Microtubules are hollow tubes made of parallel protofilaments formed by the head-to-tail assembly of αβ-tubulin heterodimers (tubulin). As a result, microtubules are polar structures, with a (−) end where α-tubulin subunits are exposed, and a faster growing (+) end, terminated by β-tubulin subunits (Desai and Mitchison, 1997). Our understanding of microtubule dynamics and of its regulation is still incomplete, in particular from a structural point of view, although continuous progress has been made over the past 2 decades. Indeed, microtubule structures are now available at near 3 Å resolution from cryo-electron microscopy data (Benoit et al., 2018; Howes et al., 2017; Zhang et al., 2015, 2018). In addition, crystal structures of non-microtubular tubulin have been obtained despite the notorious difficulty to crystallize this protein, which is related to its propensity to self-assemble into heterogeneous species. Two general strategies have been pursued to circumvent this limitation. In one of them, mutations that diminish longitudinal contacts between tubulin molecules have been introduced to disfavor self-assembly (Johnson et al., 2011). This tubulin mutant has been crystallized in complex with TOG domain proteins (Ayaz et al., 2012, 2014). The second approach is based on proteins that make well-defined complexes with tubulin, unable to assemble further. These proteins are either vertebrate stathmin-like domain proteins (SLDs) that form with tubulin a 2:1 tubulin:SLD assembly (T_2SLD) (Jourdain et al., 1997) or artificial Designed Ankyrin Repeat Proteins (DARPins) (Plückthun, 2015) selected to bind β-tubulin (Pecqueur et al., 2012), and high-resolution crystal structures of tubulin have been obtained with SLDs or with DARPins used as crystallization chaperones (Ahmad et al., 2016; Mignot et al., 2012; Nawrotek et al., 2011). These proteins have also proven useful to study the mechanism of microtubule-associated proteins (MAPs) that interact with tubulin, both structurally (Cao et al., 2014; Gigant et al., 2013; Prota et al., 2013b; Wang et al., 2017) and biochemically (Gigant et al., 2014; Li et al., 2015). However, both SLDs and DARPins may compete with MAPs for tubulin binding. Indeed, SLDs target a tubulin surface that corresponds to the exterior of the microtubule (Gigant et al., 2000), where the binding sites of numerous MAPs are clustered (Nogales and Kellogg, 2017). Competition with DARPins has also been reported (Nawrotek et al., 2014; Sharma et al., 2016). Therefore, there is a need to expand the tools available to study microtubules with proteins that bind tubulin differently from SLDs or from the DARPins used so far. In particular, only a few molecules that stabilize tubulin without interacting with its β subunit have been described (e.g., Clément et al., 2005; Wang et al., 2012).

We present here the selection and characterization of αReps that target the tubulin α subunit. αReps are artificial proteins based on a consensus sequence of a HEAT-like repeated motif

This is an open access article under the CC BY license (http://creativecommons.org/licenses/by/4.0/).

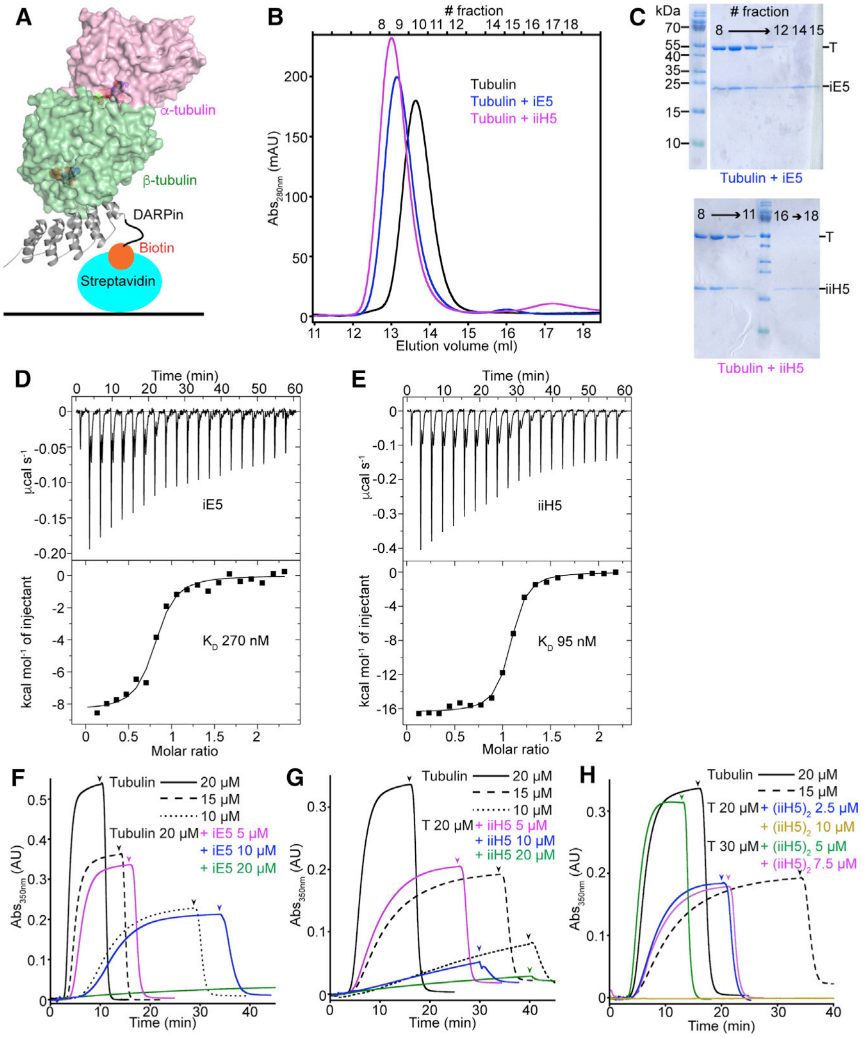

Figure 1. The iE5 and iiH5 αReps Bind Tubulin and Inhibit Microtubule Assembly

(A) Strategy for the selection of α-tubulin-specific αReps. A biotinylated version of the β-tubulin-specific DARPin A-C2 (Ahmad et al., 2016) was trapped on a streptavidin-coated plate, making the α subunit of bound tubulin most exposed.

(B) Gel filtration profile of 20 μM tubulin alone or in presence of 40 μM of either iE5 or iiH5. See also Figure S3A.

(C) Fractions defined at the top of (B) were submitted to SDS-PAGE, which confirms the formation of tubulin-αRep complexes. Irrelevant lanes have been removed from the upper gel. T, tubulin.

(D and E) ITC analysis of the interaction between tubulin and iE5 (D) or iiH5 (E). Experiments were performed by stepwise titration of the αRep (160 μM concentration) into 15 μM tubulin. Upper panels display raw data; lower panels show the integrated heat changes and associated curve fits, from which the indicated K_D values were extracted.

(F and G) iE5 and iiH5 inhibit microtubule assembly in a dose-dependent manner. The assembly of 20 μM tubulin in presence of increasing concentrations of iE5 (F) or iiH5 (G), as indicated, is compared with the assembly of 10, 15, and 20 μM tubulin alone. Microtubule assembly was monitored by turbidity. The temperature was switched from 5 to 37°C after 1 min of recording time in each case, and the arrowhead indicates the reverse temperature switch. In the case of iiH5, the assembly buffer was supplemented with 75 mM KCl to avoid aggregation.

(H) The $(iiH5)_2$ tandem repeat αRep (see Figure S2) inhibits microtubule assembly. The assembly of tubulin (20 or 30 μM) in presence of $(iiH5)_2$ at the indicated concentrations was monitored by turbidity in the conditions used in (G), from which the tubulin control curves are taken.

initially observed in thermophilic microorganisms (Guellouz et al., 2013; Urvoas et al., 2010). We show that selected αReps prevent microtubule assembly with a specific blocking effect at the (−) end, and we have determined their structure in complex with tubulin to rationalize this inhibition. These tubulin-binding αReps broaden the range of tools available to study tubulin, in particular its regulation by β-tubulin-specific proteins.

RESULTS AND DISCUSSION

Selection of α-Tubulin-Specific αReps

The *in vitro* selection of binders from a library of artificial proteins is usually performed on an immobilized target. In the case of a protein target, to preserve its native structure, this step often takes advantage of tags (e.g., a biotinylated tag that interacts with immobilized streptavidin) (Guellouz et al., 2013). However, whereas systems to express recombinant tubulin are now available (Johnson et al., 2011; Minoura et al., 2013; Ti et al., 2016; Vemu et al., 2016), purification of this protein from natural sources is still the most efficient way to obtain the large quantities needed for biochemical experiments. We therefore decided to use for selection the same protein, purified from sheep brain, that will be used in later experiments. To bias the selection toward α-tubulin binders, we immobilized a β-tubulin-specific biotinylated DARPin on a streptavidin-coated plate (Figure 1A). In addition, to increase the residence time of tubulin on the plate, we used a high-affinity, slowly dissociating DARPin (Ahmad et al., 2016). An αRep library (Guellouz et al., 2013) was then screened through three rounds of phage display, and αReps that bind tubulin were identified in an ELISA assay. Two αReps, named iE5 and iiH5, which were among those giving the highest signal in this assay, and which comprise five and three internal repeats, respectively, were chosen for further biochemical and structural characterization.

The iE5 and iiH5 αReps Bind Tubulin and Inhibit Microtubule Assembly

In the ELISA assay, the interaction of the αReps with tubulin was monitored while the latter was immobilized (Figure 1A). To ascertain the interaction in solution, we performed size exclusion chromatography experiments (Figure 1B). Compared with tubulin alone, a chromatographic peak that eluted earlier was observed when tubulin:αRep samples were loaded on the column. SDS-PAGE analysis of the protein content of that peak indicated the

Table 1. Thermodynamic Binding Parameters Determined by ITC

αReps	n	K_D (nM)	ΔH (kcal mol^{-1})	TΔS (kcal mol^{-1})	ΔG (kcal mol^{-1})
iE5	0.8	270 ± 75	−8	−0.4	−8.4
iiH5	1	95 ± 15	−16	7	−9

Table 2. Data Collection and Refinement Statistics

	Tubulin-iE5	Tubulin-iiH5
Data Collection[a]		
Space group	$P3_221$	C2
Cell dimensions		
a, b, c (Å)	102.3, 102.3, 216.2	450.8, 53.8, 229.6
α, β, γ (°)	90.0, 90.0, 120.0	90.0, 118.8, 90.0
Resolution (Å)	46.2–2.60 (2.69–2.60)	36.8–3.20 (3.31–3.20)
R_{meas}	0.169 (1.95)	0.321 (1.05)
I/σI	14.6 (1.2)	4.17 (1.02)
$CC_{1/2}$	0.999 (0.446)	0.954 (0.569)
Completeness	99.9 (100)	98.9 (98.2)
Multiplicity	13.2 (12.5)	3.2 (3.3)
Refinement		
Resolution (Å)	46.2–2.60	36.85–3.20
No. of reflections	41,238	80,684
Rwork/Rfree	0.173/0.223	0.230 (0.270)
Number of non-hydrogen atoms		
Protein	8202	23,796
Ligands	100	183
Solvent	176	0
B factors		
Protein	70.6	90.6
Ligands	75.3	89.4
Solvent	60.2	
Coordinate error (Å)	0.31	0.61
RMSD		
Bond lengths (Å)	0.010	0.010
Bond angles (°)	1.16	1.20
Ramachandran (%)		
Favored region	97.15	94.03
Allowed region	2.66	4.98
Outliers	0.19	0.99

[a]Data were collected on a single crystal. Values in parentheses are for the highest-resolution shell.

presence of both tubulin and either of the αReps (Figure 1C). These results confirm that both iE5 and iiH5 form a complex with tubulin. In addition, because the injected samples were prepared with a slight molar excess of αRep, and because a peak corresponding to free αReps (not bound to tubulin) was detected (Figure 1B), the size exclusion chromatography experiments suggest that the stoichiometry of binding is one tubulin molecule for one αRep in both cases.

The gel filtration profile is characteristic of a tight interaction. For both αReps, the peak of the complex was nearly symmetrical, and the tubulin peak was completely displaced. To characterize the strength of the association of tubulin with iE5 and iiH5 further, we studied the tubulin:αRep interaction by isothermal titration calorimetry (ITC). The titration of tubulin by iE5 led to a dissociation constant (K_D) of 270 ± 75 nM, whereas the same experiment with iiH5 led to a K_D of 95 ± 15 nM (Figures 1D and 1E; Table 1). These values are within the range usually found between selected αReps and their target protein (Chevrel et al., 2018; Guellouz et al., 2013) and correspond to reasonably tight interactions.

Then we recorded the effect of iE5 and iiH5 on microtubule assembly using a turbidity assay. We found that the turbidity signal corresponding to microtubule assembly decreased in presence of both αReps (Figures 1F and 1G). These experiments further supported the 1:1 tubulin:αRep binding stoichiometry, in agreement with the gel filtration analysis (Figure 1B) and the ITC data (Table 1). For instance, the turbidity plots of 20 μM tubulin in presence of 5 μM iE5 (Figure 1F) or iiH5 (Figure 1G) are similar to the ones of the 15 μM tubulin control. The same applies when comparing a 10-μM tubulin solution and samples consisting of 20 μM tubulin and 10 μM αRep. Finally, when a stoichiometric amount of αRep was added to 20 μM tubulin, almost no turbidity signal was detected. Taken together, these results show that both αReps inhibit microtubule assembly in a dose-dependent manner. To elucidate the basis of this mechanism, we determined the structure of the corresponding tubulin-αRep complexes.

iE5 and iiH5 Target the Longitudinal Surface of α-Tubulin

The X-ray structure of tubulin-iE5 was determined by molecular replacement at a resolution of 2.6 Å (Table 2). The structure confirmed the 1:1 tubulin:iE5 stoichiometry (Figure 2A) and there was one complex per asymmetric unit. In agreement with the selection strategy (Figure 1A), the αRep binds to α-tubulin. It targets a mostly acidic surface (Figure 2B) that is involved in tubulin-tubulin longitudinal contacts within microtubules (Nogales et al., 1999) (Figure 2C). It interacts in particular with the α-tubulin T7 loop and the following H8 helix, and with the H10-S9 loop and the S9 β-strand (Figures 2A and 2D) (see (Löwe et al., 2001) and Figure S1 for tubulin secondary structure nomenclature and domain definition). On the αRep side, the binding surface is electropositive (Figure 2D) and formed by many residues from randomized positions but also by some (invariant) residues of the framework (Figure 2E), as commonly observed in αRep selection (Guellouz et al., 2013).

The structure of tubulin-iiH5 was similarly determined to 3.2 Å resolution (Table 2, Figure 3A). There are three, virtually identical, complexes in the asymmetric unit (pairwise root-mean-square deviations (RMSD) ranging from 0.39 to 0.50 Å; approximately 1010 Cαs compared). In the crystal, tubulin-iiH5 formed a helical structure with six complexes per turn and a pitch of 54 Å (i.e., the width of one tubulin) (Figure 3B). Several features of the tubulin-iE5 structure also apply to tubulin-iiH5. Indeed, iiH5 makes a 1:1 assembly with tubulin. It binds to the (acidic) longitudinal surface of the α subunit (Figure 3C). It interacts in

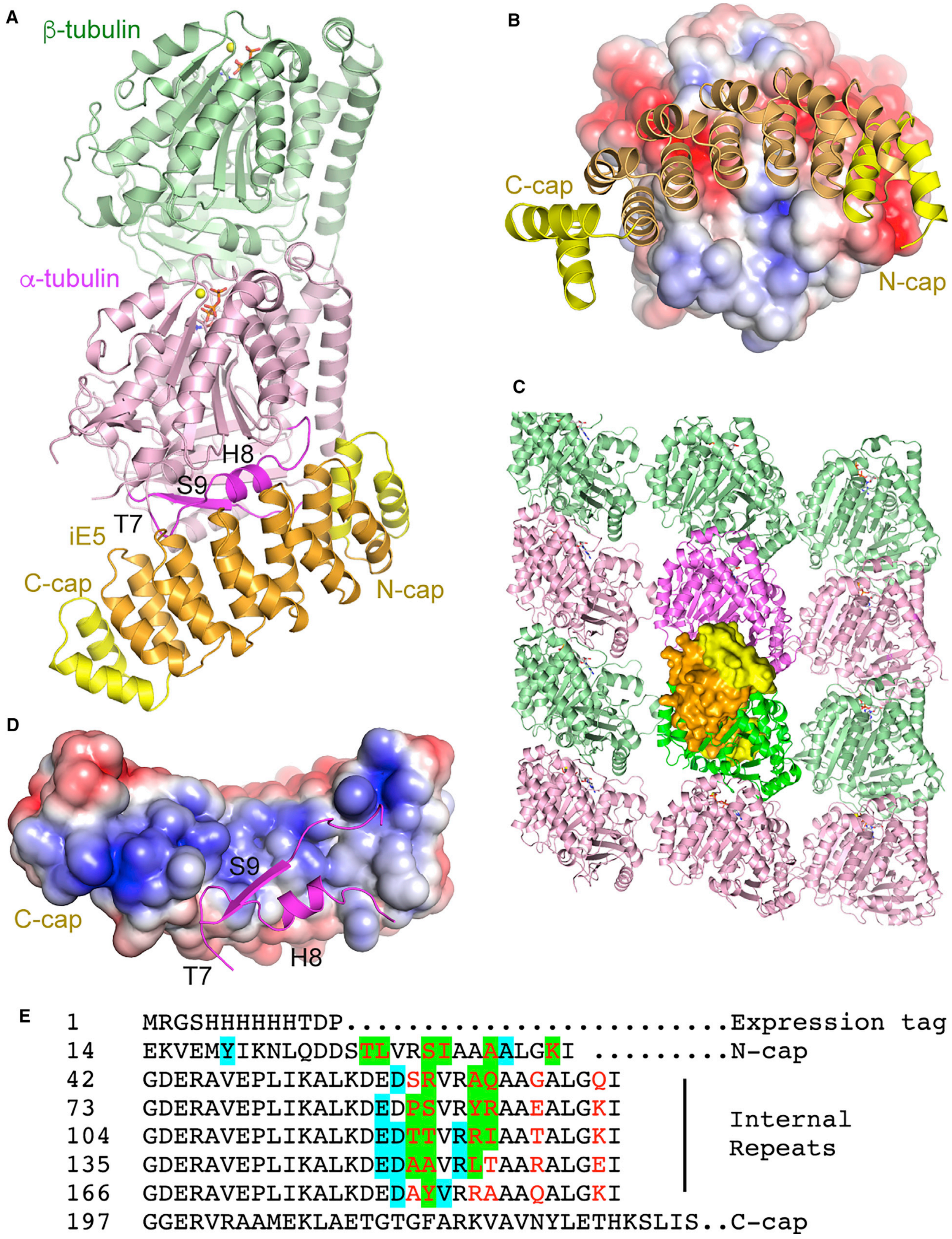

(legend on next page)

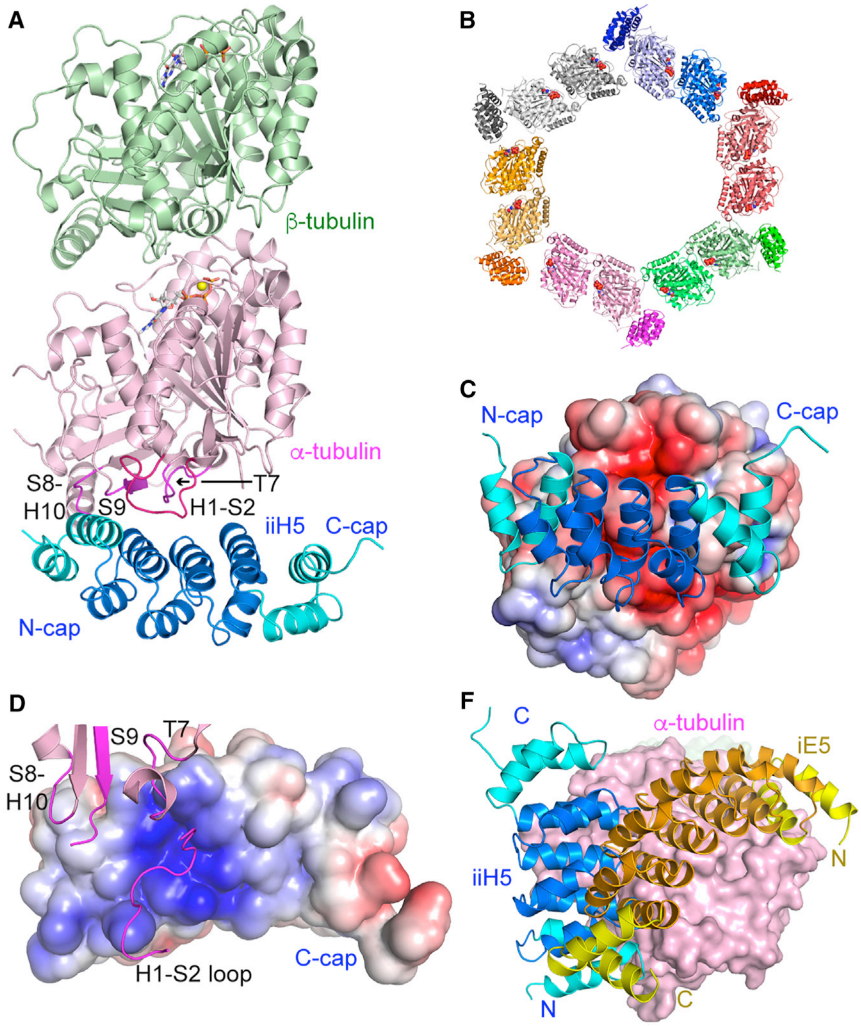

E

```
1     MRGSHHHHHHTDP........................Expression tag
14    EKVEMYIKNLQDDSPPVRFNAAVALGKI ........N-cap
42    GDERAVEPLIKALKDEDWQVRKTAAYALGKI   |   Internal
73    GDERAVEPLIKALKDEDRYVRSRAALALGKI   |   Repeats
104   GDERAVEPLIKALKDEDEYVRLSAASALGKI   |
135   GGERVRAAMEKLAETGTGFARKVAVNYLETHKSLIS..C-cap
```

Figure 3. The Tubulin-iiH5 Structure

(A) Overview of the complex crystallized.

(B) Tubulin-iiH5 forms a helical assembly of six complexes per turn in the crystal.

(C) Electrostatic potential surface of tubulin, with bound iiH5 shown as a cartoon model.

(D) Electrostatic potential surface of iiH5, with the α-tubulin elements that interact with iiH5 shown in magenta.

(E) Sequence of iiH5. See Figure 2E for color code explanations.

(F) Comparison of the tubulin-binding modes of iE5 and iiH5 αReps. The α subunit from tubulin-iiH5 has been superimposed to that from tubulin-iE5; only the latter is shown.

particular with the T7 and the S8-H10 loops and with the S9 strand (Figures 3A and 3D). iiH5 also interacts with the N-terminal H1-S2 loop. In addition, the iiH5 binding surface is basic (Figure 3D) and is mostly formed by residues at randomized positions (Figure 3E). The binding to the longitudinal surface of α-tubulin, which is exposed at the microtubule (−) end (Figure S2), suggests that these αReps may affect the two ends of the microtubule differently.

iE5, iiH5, and a Tandem Repeat αRep Stop Growth at the Microtubule (–) End

To discriminate between effects the αReps have on the growth of the two different microtubule ends, we imaged individual microtubules using a total internal reflection fluorescence microscopy (TIRFM) assay (Roostalu et al., 2015), in which dynamic microtubules grew in the presence of 15 μM tubulin from immobilized GMPCPP-microtubule "seeds". In the absence of αReps, microtubule (+) and (−) ends elongated with speeds of ~20 nm s^{-1} and 4 nm s^{-1}, respectively (Figure 4). The addition of 1 μM of iE5 (Figures 4C and 4H) or of iiH5 (Figures 4E and 4I) substantially reduced the (−) end growth speed, whereas the (+) end growth speed was unaffected. To test if this selective inhibitory effect of (−) end growth can be increased, we constructed a tandem repeat version of the iiH5 αRep (Figure S2), termed $(iiH5)_2$, as it was done previously with a β-tubulin targeting DARPin (Pecqueur et al., 2012). We first verified using a turbidity assay that the inhibition of microtubule assembly by $(iiH5)_2$ (Figure 1H) agrees with the formation of a 2:1 tubulin:$(iiH5)_2$ complex

Figure 2. The Tubulin-iE5 Structure

(A) Overview of the complex crystallized. The iE5 internal repeats are in orange, and the N-cap and C-cap are in yellow. The α-tubulin secondary structural elements (defined in Figure S1) that interact with iE5 are in magenta.

(B) Electrostatic potential surface of tubulin, with bound iE5 shown as a cartoon model.

(C) iE5 prevents inter-tubulin longitudinal interactions. iE5 (surface representation) has been modeled on a microtubule α subunit (magenta) after superposition of α-tubulin from tubulin-iE5. iE5 would clash with the β subunit (bright green) of a neighboring tubulin along a protofilament. View from the outside of the microtubule (PDB: 3JAK [Zhang et al., 2015]; two tubulin segments of three protofilaments are traced).

(D) Electrostatic potential surface of iE5, with the interacting α-tubulin elements shown in magenta.

(E) Sequence of iE5. The residues at randomized positions are in red. The residues that are less than 5 Å distant from tubulin residues in the complex are highlighted in cyan (invariant residues) or in green (randomized positions).

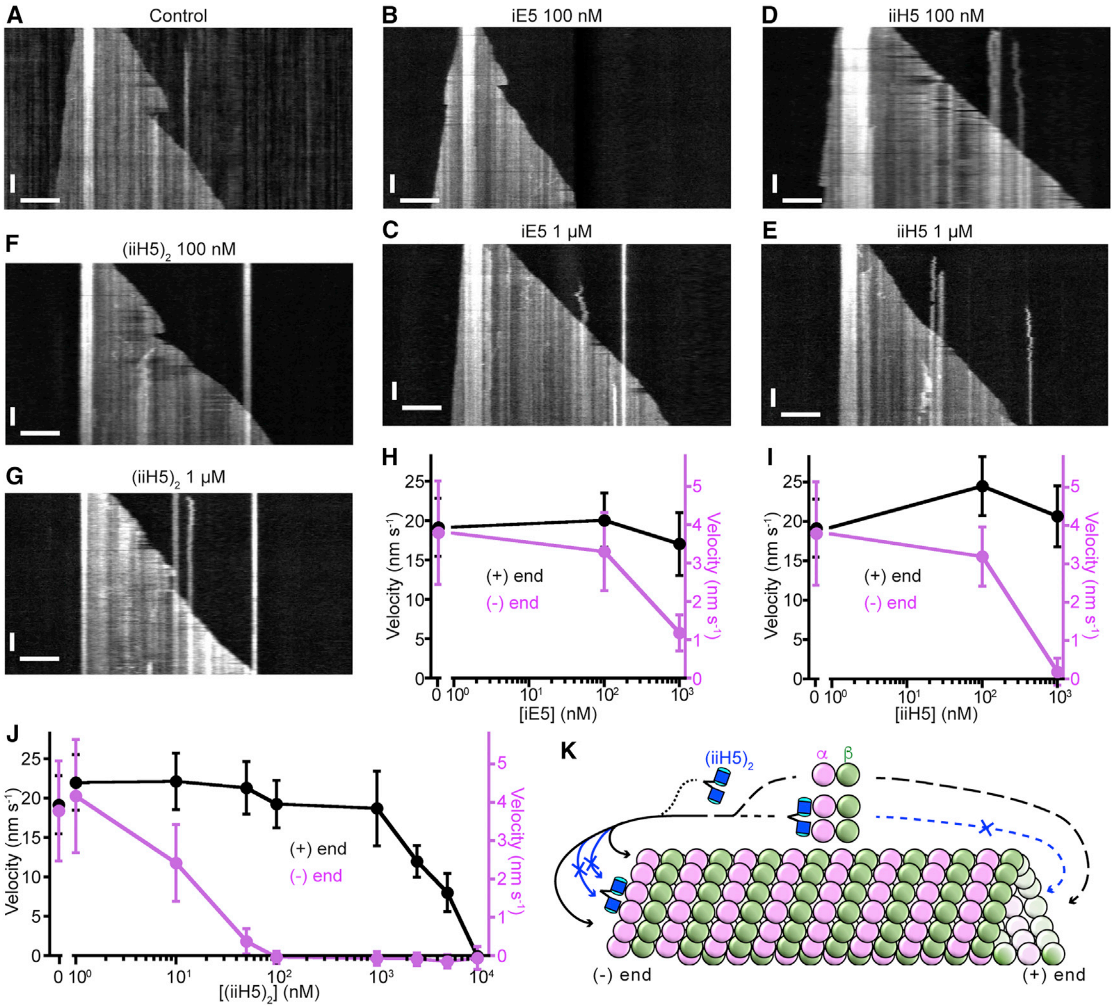

Figure 4. The αReps Selectively Inhibit Microtubule (–) End Growth

(A–G) Representative TIRFM kymographs showing individual microtubules growing from surface-immobilized GMPCPP "seeds" in the absence (A) or presence of iE5 (B and C), iiH5 (D and E), and $(iiH5)_2$ (F and G) αReps at the indicated concentrations. Experiments were performed at 30°C in presence of 15 μM CF640R-labeled tubulin. Scale bars, 6 μm (horizontal), 2 min (vertical).

(H–J) Mean growth velocities of microtubule (+) and (–) ends (black and magenta symbols, respectively) as a function of iE5 (H), iiH5 (I), and $(iiH5)_2$ (J) αRep concentration. At least 20 microtubules per condition were used for growth speed measurements. Error bars are SD.

(K) Model of microtubule assembly inhibition by the $(iiH5)_2$ tandem repeat αRep. The tubulin-$(iiH5)_2$ complex is not incorporated at the (+) end, which continues growing as long as enough free tubulin is available. By contrast, $(iiH5)_2$ or the complex it forms with tubulin associates at the (–) end but then blocks addition of tubulin heterodimers to capped protofilaments.

(V.C. et al., unpublished data). TIRFM experiments then demonstrated that $(iiH5)_2$ indeed inhibited (–) end growth more efficiently than the monomeric αReps (Figures 4F, 4G, and 4J). The microtubule (–) end growth was slowed down already in the presence of only 10 nM $(iiH5)_2$ and completely blocked at 100 nM $(iiH5)_2$. Strikingly, as in the case of the monovalent αReps, the growth of the (+) end remained unaffected up to 1 μM $(iiH5)_2$. At 10 μM $(iiH5)_2$, (+) end growth finally also stopped (i.e., at a concentration about two orders of magnitude higher than that needed to block (–) end growth).

From these results, the mechanism of microtubule assembly inhibition by these αReps can be deduced (Figure 4K). Tubulin-αRep complexes cannot be incorporated at the microtubule (+) end because the longitudinal surface of the α subunit of the incoming tubulin is masked by the αRep. Therefore, at that end, the αReps act as tubulin-sequestering proteins and high αRep concentrations are required to exert an effect. In contrast, αReps may bind at the microtubule (–) end, where α-tubulin subunits are exposed. They may bind on their own but also as a complex with tubulin because the β-tubulin longitudinal surface remains accessible in this complex. In this case, the targeted protofilaments become capped and cannot elongate further. Therefore, as long as an αRep caps the protofilament (–) end, it blocks the association of many incoming tubulins (either in complex with αReps or not). This mechanism explains why the αReps interfere with microtubule growth more drastically at (–) than at (+) ends and interfere selectively with (–) end growth at lower αRep concentrations. This mechanism

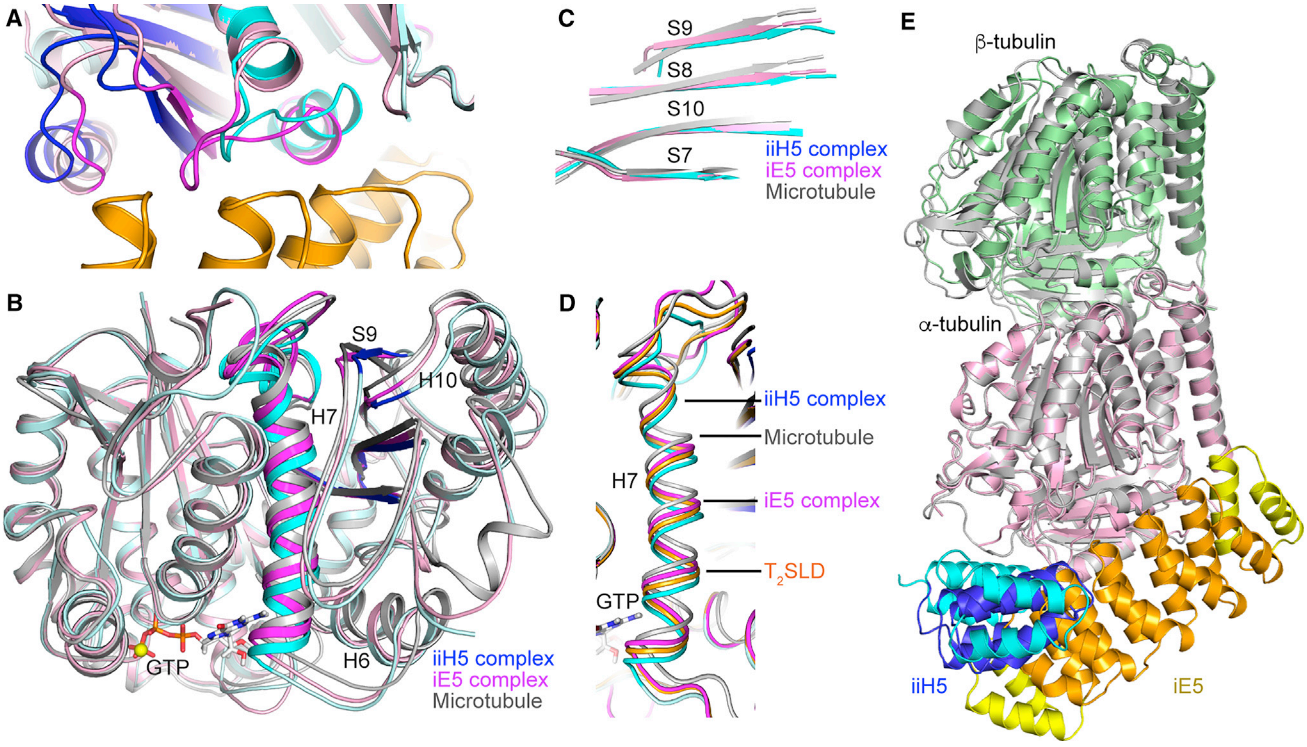

Figure 5. The α-tubulin Plasticity

(A) α-tubulin differences in the iE5 and iiH5 complexes. The α subunit of tubulin-iE5 has been superimposed to that of tubulin-iiH5, taking the secondary structural elements of the N-terminal domain as a reference (see Figure S1). α-tubulin bound to iE5 is in pink, with the regions that interact with the αRep in magenta; iE5 is in orange. α-tubulin from tubulin-iiH5 is in cyan, with the H7-T7-H8 region in brighter color and intermediate domain structural elements in blue; iiH5 is not shown. For clarity, the α-tubulin N-terminal H1-S2 loop is not traced.

(B) Comparison of α-tubulin in the iiH5 complex (cyan and blue), in tubulin-iE5 (pink and magenta) and in the microtubule (gray; PDB: 3JAK), centered on the H7 helix. The α subunits have been aligned as in (A).

(C) Same as in (B), but only the α-tubulin intermediate domain β sheet is depicted.

(D) Comparison of α-tubulin H7 position in different structures after superposition as in (A), taking tubulin-iiH5 as a reference. The comparison is with microtubular tubulin (PDB: 3JAK) and with T_2SLD (PDB: 3RYC; Nawrotek et al., 2011).

(E) Comparison of the overall conformation of αβ-tubulin bound to iiH5 (gray) and to iE5 (pink and green). After superposing the α subunits, the β subunits are misaligned by about 7°. As the tubulin β subunit is the part of this protein that is most distant from the αRep in the complexes described here, this misalignment is most likely solely due to the crystal packing.

is reminiscent of that of β-tubulin-targeting DARPins (Pecqueur et al., 2012), but with reverse outcomes at both ends of the microtubule.

The Plasticity of α-Tubulin

Although the iE5 and iiH5 αReps share the same mechanism of microtubule inhibition (Figure 4) and their epitopes on tubulin overlap, the binding modes of the two αReps also clearly differ (Figure 3F). One consequence was the possibility to engineer $(iiH5)_2$ (Figure S2), whereas the design of an iE5-based tandem repeat αRep would have been more difficult. The different binding modes also result in an overall surface area buried upon complex formation of about 1650 Å^2 in the case of tubulin-iiH5 vs about 2470 Å^2 in the case of tubulin-iE5. Interestingly, this larger buried surface does not translate into a higher affinity (Figures 1D and 1E). A tubulin conformational change might explain this apparent discrepancy (Kastritis et al., 2011). Indeed, in the complex with iE5, a different conformation of the α-tubulin T7 loop, which interacts with this αRep, is observed. This structural variation propagates to the adjacent H7 and H8 helices (Figure 5A), while remaining compatible with the binding to tubulin of, e.g., kinesin-1 and colchicine (Figure S3). The α-tubulin structural change is best pictured by comparing the H7 central helix, which translates when tubulin switches from a straight microtubular conformation to a curved soluble one (Ravelli et al., 2004). After superposition of the secondary structural elements of the N-terminal domain, a translation of about 1 Å is needed to superimpose the α subunit H7 helices of tubulin-iiH5 and tubulin-iE5, which is about half of the translation value when comparing the iiH5 complex and the microtubule (Figure 5B). This translation is accompanied by changes in the intermediate domain (Figure 5C). When the comparison is extended to other structures of non-microtubular tubulin, additional positions of the H7 helix that are intermediate between the ones in tubulin-iiH5 and tubulin-iE5 are found (Figure 5D). Therefore, the α subunit in tubulin-iE5 is in a conformation that is on the way to the ones observed in the microtubule.

Table 3. Angle between the α and β Subunits in a Subset of Tubulin Structures

	Angle Value[a]	PDB id
Microtubule	1.1°	3JAK
Tubulin–kinesin–DARPin	9.2°	4HNA
Tubulin–SLD–TTL	10.5°	4I4T
Tubulin–SLD	10.6°	3RYC
Tubulin–SLD–DARPin	10.6°	4F6R
Tubulin–iiH5[b]	11.2°	6GWD[c]
Tubulin–kinesin–DARPin	11.6°	4LNU
Tubulin–DARPin	11.9°	4DRX
Tubulin–TOG	12.2°	4U3J
Tubulin–TOG	13.5°	4FFB
Tubulin–DARPin	13.5°	5EYP
Tubulin–CPAP–DARPin	14.4°	5ITZ
Tubulin–kinesin–DARPin	14.7°	5MIO
Tubulin–iE5	18.2°	6GWC[c]

[a]Obtained by superposing the secondary structural elements of the N-terminal domain of α-tubulin to those of β-tubulin, as defined in Figure S1.
[b]Average value for the three molecules of the asymmetric unit.
[c]This work.

We then questioned whether the structural differences within the α subunit in the complexes with αReps extend to the overall conformation of tubulin. In both complexes, tubulin is in a curved conformation. We calculated angles between the α and β subunits ranging from 10.7° to 12° for the three molecules of the asymmetric unit in the complex with iiH5. In the case of tubulin-iE5, the angle is slightly larger (about 18°), being in the upper range of values found in crystal structures of tubulin (Figure 5E, Table 3). Therefore, whereas tubulin has a straight conformation in the microtubule core (Nogales et al., 1999; Zhang et al., 2015) and adopts intermediate shapes at microtubule ends (Atherton et al., 2017; Chrétien et al., 1999; Guesdon et al., 2016), the structural results presented here agree with the general view that tubulin is curved when disassembled (Gigant et al., 2000; Melki et al., 1989), with a curvature angle that is at least about 10° (Table 3). Interestingly, although the α subunit in tubulin-iE5 is in a conformation intermediate between that seen in tubulin-iiH5 and the microtubular ones, this complex displays the largest tubulin curvature. This observation suggests that, outside the microtubule context, conformational changes within the subunits are uncorrelated to the variation of the αβ-tubulin curvature.

Conclusion

In this work, we have selected α-tubulin-specific αReps. These binders prevent tubulin self-association by targeting a surface that is involved in longitudinal interactions in tubulin assemblies, with different implications for the two microtubule ends (Figure 4). Their binding mode is reminiscent of that of the N-terminal β-hairpin of SLDs (Clément et al., 2005; Wang et al., 2012), which also interacts with this tubulin surface (Ravelli et al., 2004). But SLDs stabilize in addition a second tubulin molecule through their C-terminal helix to form a T_2SLD complex (Gigant et al., 2000). Different from this case, the binding site of iE5 and iiH5 αReps is restricted to the α-tubulin longitudinal surface. Therefore, when bound to tubulin, they leave the surface that corresponds to the exterior of the microtubule accessible (Nogales et al., 1999). We anticipate that these α-tubulin-specific αReps will be useful for mechanistic and structural studies of microtubule dynamics and of tubulin:MAPs interactions, and complementary to DARPins that target the β subunit (Pecqueur et al., 2012).

Finally, our results enlighten the plasticity of the tubulin subunits. Interestingly, in microtubules, the α subunit undergoes the most substantial structural variations associated with GTP hydrolysis (Manka and Moores, 2018; Zhang et al., 2015, 2018). Our data indicate that a conformational change of α-tubulin toward the microtubule structure may be initiated outside the microtubule context. However, the full microtubular conformation has been seen only in microtubules and related assemblies (Löwe et al., 2001; Zhang et al., 2015) and remains to be captured in soluble tubulin complexes.

STAR★METHODS

Detailed methods are provided in the online version of this paper and include the following:

- KEY RESOURCES TABLE
- CONTACT FOR REAGENT AND RESOURCE SHARING
- EXPERIMENTAL MODEL AND SUBJECT DETAILS
  - αRep Library
  - Bacteria Strains
- METHOD DETAILS
  - αRep Selection
  - Protein Purification
  - Size Exclusion Chromatography
  - Isothermal Titration Calorimetry
  - Microtubule Assembly Inhibition
  - Total Internal Reflection Fluorescence Microscopy
  - Crystallization and Structure Determination
- QUANTIFICATION AND STATISTICAL ANALYSIS
- DATA AND SOFTWARE AVAILABILITY

SUPPLEMENTAL INFORMATION

Supplemental Information includes three figures and can be found with this article online at https://doi.org/10.1016/j.str.2018.12.001.

ACKNOWLEDGMENTS

We thank M. Knossow (I2BC, Gif-sur-Yvette) for discussions and critical reading of the manuscript. Diffraction data were collected at the SOLEIL synchrotron (PX1 and PX2 beam lines, Saint-Aubin, France) and at the European Synchrotron Radiation Facility (ID30 beam line, Grenoble, France). We are most grateful to the machine and beam line groups for making these experiments possible. We thank D. Mauchand (Unité Commune d'Expérimentation Animale, Institut National de la Recherche Agronomique, Jouy en Josas, France) for providing us with the material from which tubulin was purified. This work has benefited from the facilities and expertise of the I2BC crystallization and biophysics platforms. This work has been supported by the Fondation ARC pour la Recherche sur le Cancer (to B.G.), by CNRS, and by the French Infrastructure for Integrated Structural Biology (FRISBI) ANR-10-INSB-05-01. T.C. and T.S. were supported by the Francis Crick Institute, which receives its core funding from Cancer Research UK (FC001163), the UK

Medical Research Council (FC001163), and the Wellcome Trust (FC001163), and by the European Research Council (Advanced Grant, project 323042).

AUTHOR CONTRIBUTIONS

P.M. and B.G. designed research; V.C., A.U., S.C.F., and M.A.N. performed research; V.C., A.U., M.V.L., P.M., and B.G. analyzed data; T.C. and T.S. designed and performed the TIRFM experiments; B.G. wrote the manuscript with input from all authors.

DECLARATION OF INTERESTS

The authors declare no competing interests.

Received: July 26, 2018
Revised: October 24, 2018
Accepted: December 3, 2018
Published: January 17, 2019

REFERENCES

Ahmad, S., Pecqueur, L., Dreier, B., Hamdane, D., Aumont-Nicaise, M., Plückthun, A., Knossow, M., and Gigant, B. (2016). Destabilizing an interacting motif strengthens the association of a designed ankyrin repeat protein with tubulin. Sci. Rep. *6*, 28922.

Atherton, J., Jiang, K., Stangier, M.M., Luo, Y., Hua, S., Houben, K., van Hooff, J.J.E., Joseph, A.P., Scarabelli, G., Grant, B.J., et al. (2017). A structural model for microtubule minus-end recognition and protection by CAMSAP proteins. Nat. Struct. Mol. Biol. *24*, 931–943.

Ayaz, P., Munyoki, S., Geyer, E.A., Piedra, F.A., Vu, E.S., Bromberg, R., Otwinowski, Z., Grishin, N.V., Brautigam, C.A., and Rice, L.M. (2014). A tethered delivery mechanism explains the catalytic action of a microtubule polymerase. Elife *3*, e03069.

Ayaz, P., Ye, X., Huddleston, P., Brautigam, C.A., and Rice, L.M. (2012). A TOG:$\alpha\beta$-tubulin complex structure reveals conformation-based mechanisms for a microtubule polymerase. Science *337*, 857–860.

Baker, N.A., Sept, D., Joseph, S., Holst, M.J., and McCammon, J.A. (2001). Electrostatics of nanosystems: application to microtubules and the ribosome. Proc. Natl. Acad. Sci. U S A *98*, 10037–10041.

Benoit, M., Asenjo, A.B., and Sosa, H. (2018). Cryo-EM reveals the structural basis of microtubule depolymerization by kinesin-13s. Nat. Commun. *9*, 1662.

Bieling, P., Telley, I.A., Hentrich, C., Piehler, J., and Surrey, T. (2010). Fluorescence microscopy assays on chemically functionalized surfaces for quantitative imaging of microtubule, motor, and +TIP dynamics. Methods Cell Biol. *95*, 555–580.

Bricogne, G., Blanc, E., Brandl, M., Flensburg, C., Keller, P., Paciorek, W., Roversi, P., Sharff, A., Smart, O.S., Vonrhein, C., et al. (2017). BUSTER Version 2.10.3 (Global Phasing Ltd).

Cao, L., Wang, W., Jiang, Q., Wang, C., Knossow, M., and Gigant, B. (2014). The structure of apo-kinesin bound to tubulin links the nucleotide cycle to movement. Nat. Commun. *5*, 5364.

Castoldi, M., and Popov, A.V. (2003). Purification of brain tubulin through two cycles of polymerization-depolymerization in a high-molarity buffer. Protein Expr. Purif. *32*, 83–88.

Chevrel, A., Mesneau, A., Sanchez, D., Celma, L., Quevillon-Cheruel, S., Cavagnino, A., Nessler, S., Li de la Sierra-Gallay, I., van Tilbeurgh, H., Minard, P., et al. (2018). Alpha repeat proteins (αRep) as expression and crystallization helpers. J. Struct. Biol. *201*, 88–99.

Chrétien, D., Jainosi, I., Taveau, J.C., and Flyvbjerg, H. (1999). Microtubule's conformational cap. Cell Struct. Funct. *24*, 299–303.

Clément, M.J., Jourdain, I., Lachkar, S., Savarin, P., Gigant, B., Knossow, M., Toma, F., Sobel, A., and Curmi, P.A. (2005). N-terminal stathmin-like peptides bind tubulin and impede microtubule assembly. Biochemistry *44*, 14616–14625.

Desai, A., and Mitchison, T.J. (1997). Microtubule polymerization dynamics. Annu. Rev. Cell Dev. Biol. *13*, 83–117.

Dorléans, A., Knossow, M., and Gigant, B. (2007). Studying drug-tubulin interactions by X-ray crystallography. Methods Mol. Med. *137*, 235–243.

Duellberg, C., Trokter, M., Jha, R., Sen, I., Steinmetz, M.O., and Surrey, T. (2014). Reconstitution of a hierarchical +TIP interaction network controlling microtubule end tracking of dynein. Nat. Cell Biol. *16*, 804–811.

Emsley, P., Lohkamp, B., Scott, W.G., and Cowtan, K. (2010). Features and development of coot. Acta Crystallogr. D Biol. Crystallogr. *66*, 486–501.

Gasteiger, E., Hoogland, C., Gattiker, A., Duvaud, S., Wilkins, M.R., Appel, R.D., and Bairoch, A. (2005). Protein identification and analysis tools on the ExPASy server. In The Proteomics Protocols Handbook, J.M. Walker, ed. (Humana Press), pp. 571–607.

Gigant, B., Curmi, P.A., Martin-Barbey, C., Charbaut, E., Lachkar, S., Lebeau, L., Siavoshian, S., Sobel, A., and Knossow, M. (2000). The 4 Å X-ray structure of a tubulin:stathmin-like domain complex. Cell *102*, 809–816.

Gigant, B., Landrieu, I., Fauquant, C., Barbier, P., Huvent, I., Wieruszeski, J.M., Knossow, M., and Lippens, G. (2014). Mechanism of Tau-promoted microtubule assembly as probed by NMR spectroscopy. J. Am. Chem. Soc. *136*, 12615–12623.

Gigant, B., Wang, W., Dreier, B., Jiang, Q., Pecqueur, L., Pluckthun, A., Wang, C., and Knossow, M. (2013). Structure of a kinesin-tubulin complex and implications for kinesin motility. Nat. Struct. Mol. Biol. *20*, 1001–1007.

Guellouz, A., Valerio-Lepiniec, M., Urvoas, A., Chevrel, A., Graille, M., Fourati-Kammoun, Z., Desmadril, M., van Tilbeurgh, H., and Minard, P. (2013). Selection of specific protein binders for pre-defined targets from an optimized library of artificial helicoidal repeat proteins (alphaRep). PLoS One *8*, e71512.

Guesdon, A., Bazile, F., Buey, R.M., Mohan, R., Monier, S., Garcia, R.R., Angevin, M., Heichette, C., Wieneke, R., Tampe, R., et al. (2016). EB1 interacts with outwardly curved and straight regions of the microtubule lattice. Nat. Cell Biol. *18*, 1102–1108.

Howes, S.C., Geyer, E.A., LaFrance, B., Zhang, R., Kellogg, E.H., Westermann, S., Rice, L.M., and Nogales, E. (2017). Structural differences between yeast and mammalian microtubules revealed by cryo-EM. J. Cell Biol. *216*, 2669–2677.

Hyman, A., Drechsel, D., Kellogg, D., Salser, S., Sawin, K., Steffen, P., Wordeman, L., and Mitchison, T. (1991). Preparation of modified tubulins. Methods Enzymol. *196*, 478–485.

Johnson, V., Ayaz, P., Huddleston, P., and Rice, L.M. (2011). Design, overexpression, and purification of polymerization-blocked yeast $\alpha\beta$-tubulin mutants. Biochemistry *50*, 8636–8644.

Jourdain, L., Curmi, P., Sobel, A., Pantaloni, D., and Carlier, M.F. (1997). Stathmin: a tubulin-sequestering protein which forms a ternary T2S complex with two tubulin molecules. Biochemistry *36*, 10817–10821.

Kabsch, W. (2010). XDS. Acta Crystallogr. D Biol. Crystallogr. *66*, 125–132.

Kastritis, P.L., Moal, I.H., Hwang, H., Weng, Z., Bates, P.A., Bonvin, A.M., and Janin, J. (2011). A structure-based benchmark for protein-protein binding affinity. Protein Sci. *20*, 482–491.

Legrand, P. (2017). XDSME: XDS made easier. GitHub. https://doi.org/10.5281/zenodo.837885.

Li, X.H., Culver, J.A., and Rhoades, E. (2015). Tau binds to multiple tubulin dimers with helical structure. J. Am. Chem. Soc. *137*, 9218–9221.

Löwe, J., Li, H., Downing, K.H., and Nogales, E. (2001). Refined structure of $\alpha\beta$-tubulin at 3.5 Å resolution. J. Mol. Biol. *313*, 1045–1057.

Manka, S.W., and Moores, C.A. (2018). The role of tubulin-tubulin lattice contacts in the mechanism of microtubule dynamic instability. Nat. Struct. Mol. Biol. *25*, 607–615.

Maurer, S.P., Cade, N.I., Bohner, G., Gustafsson, N., Boutant, E., and Surrey, T. (2014). EB1 accelerates two conformational transitions important for microtubule maturation and dynamics. Curr. Biol. *24*, 372–384.

McCoy, A.J., Grosse-Kunstleve, R.W., Adams, P.D., Winn, M.D., Storoni, L.C., and Read, R.J. (2007). Phaser crystallographic software. J. Appl. Crystallogr. *40*, 658–674.

Melki, R., Carlier, M.F., Pantaloni, D., and Timasheff, S.N. (1989). Cold depolymerization of microtubules to double rings: geometric stabilization of assemblies. Biochemistry *28*, 9143–9152.

Mignot, I., Pecqueur, L., Dorleans, A., Karuppasamy, M., Ravelli, R.B., Dreier, B., Pluckthun, A., Knossow, M., and Gigant, B. (2012). Design and characterization of modular scaffolds for tubulin assembly. J. Biol. Chem. *287*, 31085–31094.

Minoura, I., Hachikubo, Y., Yamakita, Y., Takazaki, H., Ayukawa, R., Uchimura, S., and Muto, E. (2013). Overexpression, purification, and functional analysis of recombinant human tubulin dimer. FEBS Lett. *587*, 3450–3455.

Nawrotek, A., Guimarães, B.G., Velours, C., Subtil, A., Knossow, M., and Gigant, B. (2014). Biochemical and structural insights into microtubule perturbation by CopN from *Chlamydia pneumoniae*. J. Biol. Chem. *289*, 25199–25210.

Nawrotek, A., Knossow, M., and Gigant, B. (2011). The determinants that govern microtubule assembly from the atomic structure of GTP-tubulin. J. Mol. Biol. *412*, 35–42.

Nogales, E., and Kellogg, E.H. (2017). Challenges and opportunities in the high-resolution cryo-EM visualization of microtubules and their binding partners. Curr. Opin. Struct. Biol. *46*, 65–70.

Nogales, E., Whittaker, M., Milligan, R.A., and Downing, K.H. (1999). High-resolution model of the microtubule. Cell *96*, 79–88.

Pecqueur, L., Duellberg, C., Dreier, B., Jiang, Q., Wang, C., Plückthun, A., Surrey, T., Gigant, B., and Knossow, M. (2012). A designed ankyrin repeat protein selected to bind to tubulin caps the microtubule plus end. Proc. Natl. Acad. Sci. U S A *109*, 12011–12016.

Plückthun, A. (2015). Designed ankyrin repeat proteins (DARPins): binding proteins for research, diagnostics, and therapy. Annu. Rev. Pharmacol. Toxicol. *55*, 489–511.

Prota, A.E., Bargsten, K., Zurwerra, D., Field, J.J., Diaz, J.F., Altmann, K.H., and Steinmetz, M.O. (2013a). Molecular mechanism of action of microtubule-stabilizing anticancer agents. Science *339*, 587–590.

Prota, A.E., Magiera, M.M., Kuijpers, M., Bargsten, K., Frey, D., Wieser, M., Jaussi, R., Hoogenraad, C.C., Kammerer, R.A., Janke, C., et al. (2013b). Structural basis of tubulin tyrosination by tubulin tyrosine ligase. J. Cell Biol. *200*, 259–270.

Ravelli, R.B., Gigant, B., Curmi, P.A., Jourdain, I., Lachkar, S., Sobel, A., and Knossow, M. (2004). Insight into tubulin regulation from a complex with colchicine and a stathmin-like domain. Nature *428*, 198–202.

Roostalu, J., Cade, N.I., and Surrey, T. (2015). Complementary activities of TPX2 and chTOG constitute an efficient importin-regulated microtubule nucleation module. Nat. Cell Biol. *17*, 1422–1434.

Sharma, A., Aher, A., Dynes, N.J., Frey, D., Katrukha, E.A., Jaussi, R., Grigoriev, I., Croisier, M., Kammerer, R.A., Akhmanova, A., et al. (2016). Centriolar CPAP/SAS-4 imparts slow processive microtubule growth. Dev. Cell *37*, 362–376.

Ti, S.C., Pamula, M.C., Howes, S.C., Duellberg, C., Cade, N.I., Kleiner, R.E., Forth, S., Surrey, T., Nogales, E., and Kapoor, T.M. (2016). Mutations in human tubulin proximal to the kinesin-binding site alter dynamic instability at microtubule plus- and minus-ends. Dev. Cell *37*, 72–84.

Urvoas, A., Guellouz, A., Valerio-Lepiniec, M., Graille, M., Durand, D., Desravines, D.C., van Tilbeurgh, H., Desmadril, M., and Minard, P. (2010). Design, production and molecular structure of a new family of artificial alpha-helicoidal repeat proteins (αRep) based on thermostable HEAT-like repeats. J. Mol. Biol. *404*, 307–327.

Vemu, A., Atherton, J., Spector, J.O., Szyk, A., Moores, C.A., and Roll-Mecak, A. (2016). Structure and dynamics of single-isoform recombinant neuronal human tubulin. J. Biol. Chem. *291*, 12907–12915.

Wang, W., Cantos-Fernandes, S., Lv, Y., Kuerban, H., Ahmad, S., Wang, C., and Gigant, B. (2017). Insight into microtubule disassembly by kinesin-13s from the structure of Kif2C bound to tubulin. Nat. Commun. *8*, 70.

Wang, W., Jiang, Q., Argentini, M., Cornu, D., Gigant, B., Knossow, M., and Wang, C. (2012). Kif2C minimal functional domain has unusual nucleotide binding properties that are adapted to microtubule depolymerization. J. Biol. Chem. *287*, 15143–15153.

Zhang, R., Alushin, G.M., Brown, A., and Nogales, E. (2015). Mechanistic origin of microtubule dynamic instability and its modulation by EB proteins. Cell *162*, 849–859.

Zhang, R., LaFrance, B., and Nogales, E. (2018). Separating the effects of nucleotide and EB binding on microtubule structure. Proc. Natl. Acad. Sci. U S A *115*, E6191–E6200.

STAR★METHODS

KEY RESOURCES TABLE

REAGENT or RESOURCE	SOURCE	IDENTIFIER
Antibodies		
HRPO-conjugated anti-M13 monoclonal antibody	GE Healthcare	Cat# 27-9421-01; RRID: AB_2616587
Bacterial and Virus Strains		
E. coli Bl21(DE3)STAR	ThermoFisher Scientific	http://www.thermofisher.com/fr
E. coli Bl21(DE3)	New England Biolabs	http://international.neb.com/
E. coli XL1-Blue	New England Biolabs	http://international.neb.com/
Lib2.1 αRep library in M13 phage	(Guellouz et al., 2013)	N/A
Biological Samples		
Sheep tubulin	Purified according to (Castoldi and Popov, 2003)	N/A
Porcine tubulin	Purified according to (Castoldi and Popov, 2003)	N/A
Chemicals, Peptides, and Recombinant Proteins		
InstantBlue	Expedeon	Cat# ISB1L
Crystallization screens	Qiagen	https://www.qiagen.com
Biotinylated A-C2 DARPin	This paper and (Ahmad et al., 2016)	N/A
iE5 αRep	This paper	N/A
iiH5 αRep	This paper	N/A
$(iiH5)_2$ αRep	This paper	N/A
Cys-light kinesin-1 motor domain, 1-349 construct	(Cao et al., 2014)	N/A
Deposited Data		
Crystal structure of tubulin–iE5	This paper	PDB: 6GWC
Crystal structure of tubulin–iiH5	This paper	PDB: 6GWD
Atomic coordinates	(Zhang et al., 2015)	PDB: 3JAK
Atomic coordinates	(Nawrotek et al., 2014)	PDB: 3RYC
Atomic coordinates	(Gigant et al., 2013)	PDB: 4HNA
Atomic coordinates	(Prota et al., 2013a)	PDB: 4I4T
Atomic coordinates	(Mignot et al., 2012)	PDB: 4F6R
Atomic coordinates	(Cao et al., 2014)	PDB: 4LNU
Atomic coordinates	(Pecqueur et al., 2012)	PDB: 4DRX
Atomic coordinates	(Ayaz et al., 2014)	PDB: 4U3J
Atomic coordinates	(Ayaz et al., 2012)	PDB: 4FFB
Atomic coordinates	(Ahmad et al., 2016)	PDB: 5EYP
Atomic coordinates	(Sharma et al., 2016)	PDB: 5ITZ
Atomic coordinates	(Wang et al., 2017)	PDB: 5MIO
Atomic coordinates	(Urvoas et al., 2010)	PDB: 3LTJ
Recombinant DNA		
pQE-81L	Qiagen	http://www.qiagen.com
pBirAcm	Avidity, LLC	https://www.avidity.com/
pDST67	University of Zurich, Plückthun lab	N/A
Software and Algorithms		
Origin 7.0	OriginLab	http://www.originlab.com/
ImageJ	NIH	https://imagej.nih.gov/ij/

(*Continued on next page*)

Continued		
REAGENT or RESOURCE	SOURCE	IDENTIFIER
XDS	(Kabsch, 2010)	http://xds.mpimf-heidelberg.mpg.de/
XDSME	(Legrand, 2017)	https://github.com/legrandp/xdsme
Phaser	(McCoy et al., 2007)	http://www.phaser.cimr.cam.ac.uk/index.php/Molecular_Replacement
Buster	(Bricogne et al., 2017)	https://www.globalphasing.com/buster/
Coot	(Emsley et al., 2010)	https://www2.mrc-lmb.cam.ac.uk/personal/pemsley/coot/
Pymol	Schrödinger LLC	https://pymol.org/2/
APBS	(Baker et al., 2001)	http://www.poissonboltzmann.org/
Kaleidagraph 4.5	Synergy software	http://www.synergy.com/
Other		
HisTrap HP	GE Healthcare	Cat# 17-5248-02
HiLoad 16/60 Superdex 75 pg	GE Healthcare	Cat# 17-1068-01
Superdex 200 10/300 GL	GE Healthcare	Cat# 17-5175-01

CONTACT FOR REAGENT AND RESOURCE SHARING

Further information and requests for resources and reagents should be directed to and will be fulfilled by the Lead Contact, Benoît Gigant (benoit.gigant@i2bc.paris-saclay.fr)

EXPERIMENTAL MODEL AND SUBJECT DETAILS

αRep Library

Anti-tubulin αReps were selected from the 2.1 optimized αRep library (Guellouz et al., 2013).

Bacteria Strains

XL1-Blue, Bl21(DE3) and Bl21(DE3)STAR cells were cultured in 2YT medium in the presence of appropriate antibiotics.

METHOD DETAILS

αRep Selection

αRep selection was performed by phage display essentially following published procedures (Guellouz et al., 2013). To immobilize tubulin, the gene coding for the high-affinity tubulin-binding DARPin A-C2 (Ahmad et al., 2016) was modified to introduce an AviTag biotinylation coding sequence at the C-terminal end of the protein. Modified A-C2 was expressed in *E. coli* Bl21(DE3)STAR co-transformed with the pBirAcm plasmid (Avidity, LLC, USA) for *in vivo* biotinylation and purified as described for non-biotinylated A-C2 (Ahmad et al., 2016). Tubulin was trapped through its interaction with biotinylated A-C2 that was immobilized on a streptavidin-coated plate (Figure 1A). After each round of selection, bound phages eluted either in acidic conditions or more specifically by adding DARPin or tubulin were amplified in XL1-Blue cells and used for the following selection round. After 3 rounds, individual clones were screened for tubulin binding by phage-ELISA (Guellouz et al., 2013).

Protein Purification

αRep genes were subcloned in pQE-81L plasmid (Qiagen) for expression in *E. coli* Bl21(DE3) in 2YT medium at 37°C. After sonication of the bacteria suspension, αReps were purified from the soluble fraction by Ni^{2+}-affinity chromatography (Histrap HP, GE Healthcare) followed by gel filtration (Superdex 75 16/60 HL, GE Healthcare) in 20 mM Pipes-K, pH 6.8, 1 mM $MgCl_2$, 0.5 mM EGTA and 150 mM KCl. In the case of iiH5, the storage buffer contained 500 mM KCl. The $(iiH5)_2$ tandem repeat αRep (Figure S2; Campanacci et al, submitted) was produced and purified as iiH5. The concentration of αReps was estimated by UV spectrophotometry using theoretical extinction coefficients at 280 nm (Gasteiger et al., 2005). Tubulin was purified by two cycles of assembly in a high-molarity buffer followed by disassembly (Castoldi and Popov, 2003). Sheep brain tubulin was used throughout, except for the TIRFM experiments which were performed with porcine brain tubulin. Before use, an additional cycle of assembly and disassembly was performed to remove inactive protein. To prepare the tubulin–colchicine complex used in Figure S3, colchicine was included in the disassembly buffer (Dorléans et al., 2007). The motor domain of

the human kinesin-1 Kif5B (cys-light construct, comprising residues 1 to 349) was produced and purified as described (Cao et al., 2014).

Size Exclusion Chromatography

Samples were analyzed on a Superdex 200 10/300 GL column (GE Healthcare) equilibrated with 20 mM Pipes-K, pH 6.8, 1 mM $MgCl_2$, 0.5 mM EGTA and 150 mM KCl, unless otherwise mentioned. The content of the chromatographic peaks was analyzed by SDS-PAGE with Coomassie Blue staining.

Isothermal Titration Calorimetry

Calorimetric experiments were conducted at 20°C with a MicroCal ITC200 instrument (Malvern). All proteins were buffer-exchanged to 20 mM Pipes-K pH 6.8, 1 mM $MgCl_2$, 0.01 mM EGTA, 0.01 mM GDP and 75 mM KCl. Aliquots (2 μL) of iE5 or iiH5 at 160 μM were injected into a 15 μM tubulin solution (cell volume, 0.24 mL). Analysis of the data was performed using the MicroCal Origin software provided by the manufacturer according to the one-binding-site model.

Microtubule Assembly Inhibition

Microtubule assembly was performed in a buffer consisting of 50 mM Mes-K, pH 6.8, 6 mM $MgCl_2$, 1 mM EGTA, 30% (v/v) glycerol, and 0.5 mM GTP. It was initiated by raising the temperature from 5°C to 37°C and monitored at 350 nm with a Cary 50 spectrophotometer (Agilent Technologies), using a 0.7-cm path length cuvette. In presence of iiH5 and of $(iiH5)_2$, to avoid aggregation, the assembly buffer was supplemented with 75 mM KCl.

Total Internal Reflection Fluorescence Microscopy

Tubulin was labeled with CF640R-N-hydroxysuccinimide ester (NHS, Sigma-Aldrich) or biotin-NHS ester (Thermo scientific) (Hyman et al., 1991). Flow chambers for TIRF microscopy experiments were assembled from polyethylene glycol (PEG)-passivated functionalized glass and poly(L-lysine)-PEG (SuSoS)-passivated counter glass (Bieling et al., 2010). Biotin-PEG-coated glass was prepared by mixing 91% hydroxyl-PEG-3000-amine and 9% biotin-PEG-3000-amine (both from RAPP Polymere) and coupling this mixture to glass. Fluorescently-labeled biotinylated GMPCPP-stabilized microtubule 'seeds' (containing 20% CF640R-labeled tubulin) for assays with dynamic microtubules were prepared as described (Bieling et al., 2010; Roostalu et al., 2015).

The assay was performed essentially as described earlier (Roostalu et al., 2015). In brief, flow chambers were incubated with 5% Pluronic F-127 in MQ water (Sigma-Aldrich) for 10 min at room temperature, washed with assay buffer (AB: 80 mM Pipes, 75 mM KCl, 1 mM EGTA, 1 mM $MgCl_2$, 1 mM GTP, 5 mM 2-mercaptoethanol, 0.15% (w/v) methylcellulose (4,000 cP; Sigma-Aldrich), 1% (w/v) glucose, 0.02% (v/v) Brij-35) supplemented with 50 μg mL^{-1} κ-casein (Sigma-Aldrich). Chambers were subsequently incubated with the same buffer additionally containing 50 μg mL^{-1} NeutrAvidin (Life Technologies) for 3 min on a metal block on ice, washed with AB and then incubated with AB containing an appropriate dilution of fluorescently-labeled GMPCPP-microtubule 'seeds' for 3 min at room temperature. Unbound 'seeds' were removed by additional washes with AB followed by the final assay mixture: 50% (v/v) 2x AB, 48.18% BRB80 (80 mM Pipes, 1 mM EGTA, 1 mM $MgCl_2$) supplemented with oxygen scavengers (682 μg/mL^{-1} glucose oxidase (Serva), 164 μg/mL^{-1} catalase (Sigma-Aldrich)) and 15 μM CF640R-labeled tubulin (labeling ratio: 6.5%), and 1.8% of varying concentrations of α-Reps diluted in their storage buffers. Flow chambers were sealed with vacuum grease (Beckman) and imaging was started 90 s after placing the chamber on the microscope. Experiments were performed at 30°C ± 1°C on a TIRF microscope (iMIC, FEI Munich) described in detail previously (Duellberg et al., 2014; Maurer et al., 2014). Image acquisition was carried out as described before (Duellberg et al., 2014; Maurer et al., 2014). All time-lapse videos were recorded at 1 frame per 5 s with a 200-ms exposure time. CF640R-labeled microtubules were excited at 640 nm keeping the laser power constant for all experiments. Mean microtubule growth speeds were calculated from kymographs generated using ImageJ.

Crystallization and Structure Determination

Tubulin–iE5 was crystallized at 293 K by vapor diffusion in a crystallization buffer consisting of 13% (v/v) PEG 400, 0.1 M Mes-K pH 6.8. Crystals were harvested in a mother liquor containing 20% PEG 400 and flash-cooled in liquid nitrogen. Tubulin–iiH5 crystals were obtained at 277 K in 0.2 M Na tartrate, 12% (w/v) PEG 3350 and cryoprotected in mother liquor supplemented with 20% glycerol. Datasets were collected at 100 K at the Proxima-1 beamline (SOLEIL Synchrotron, Saint-Aubin, France). Data were processed with XDS (Kabsch, 2010) using the XDSME package (Legrand, 2017). Structures were solved by molecular replacement with Phaser (McCoy et al., 2007) using tubulin (PDB: 4DRX) and αRep-n4-a (PDB: 3LTJ) as search models, and refined with BUSTER (Bricogne et al., 2017) with iterative model building in Coot (Emsley et al., 2010). Data collection and refinement statistics are reported in Table 2. Figures of structural models were generated with PyMOL (www.pymol.org). The electrostatic potential surface was calculated using APBS (Baker et al., 2001) and rendered in PyMOL.

QUANTIFICATION AND STATISTICAL ANALYSIS

Table 2 contains quantitative parameters related to data and refinement statistics. The uncertainty on the K_D determined by ITC (Table 1) was estimated by the Origin software using the Levenberg-Marquardt algorithm. Error bars in the TIRFM experiments (Figures 4H–4J) are SD from measurements of at least 20 microtubules.

DATA AND SOFTWARE AVAILABILITY

The accession numbers for the coordinates and structure factors for the tubulin-iE5 and tubulin-iiH5 crystal structures reported in this paper are PDB: 6GWC and PDB: 6GWD, respectively.

Structure, Volume *27*

Supplemental Information

Selection and Characterization of Artificial Proteins Targeting the Tubulin α Subunit

Valérie Campanacci, Agathe Urvoas, Tanja Consolati, Soraya Cantos-Fernandes, Magali Aumont-Nicaise, Marie Valerio-Lepiniec, Thomas Surrey, Philippe Minard, and Benoît Gigant

```
             S1 T1         H1                          H1′  S1′     S1"   S2 T2    H2
a-Tubulin  MRECISIHVGQAGVQIGNACWELYCLEHGIQPDGQMPSDKTIGGGDDSFNTFFSETGAGKHVPRAVFVDLEPTVIDEVRT
                    10        20        30        40        50        60        70        80
b-Tubulin  MREIVHIQAGQCGNQIGAKFWEVISDEHGIDPTGSYHGDSDLQL..ERINVYYNEATGNKYVPRAILVDLEPGTMDSVRS
                S1 T1         H1                    H1′   H1′   S1′    S1"   S2 T2    H2

              H2′  H2" S3   T3      H3′          H3                S4   T4        H4
a-Tubulin  GTYRQLFHPEQLITGKEDAANNYARGHYTIGKEIIDLVLDRIRKLADQCTGLQGFLVFHSFGGGTGSGFTSLLMERLSVD
                    90        100       110       120       130       140       150       160
b-Tubulin  GPFGQIFRPDNFVFGQSGAGNNWAKGHYTEGAELVDSVLDVVRKESESCDCLQGFQLTHSLGGGTGSGMGTLLISKIREE
              H2′  H2" S3   T3              H3                    S4    T4       H4

                  S5      T5            H5          S6 T6    H6                  H7
a-Tubulin  YGKKSKLEFSIYPAPQVSTAVVEPYNSILTTHTTLEHSDCAFMVDNEAIYDICRRNLDIERPTYTNLNRLISQIVSSITA
                    170       180       190       200       210       220       230       240
b-Tubulin  YPDRIMNTFSVMPSPKVSDTVVEPYNATLSVHQLVENTDETYCIDNEALYDICFRTLKLTTPTYGDLNHLVSATMSGVTT
                  S5      T5            H5          S6 T6    H6                  H7

            H7   T7      H8             S7     M loop         H9     H9′      H9"     S8
a-Tubulin  SLRFDGALNVDLTEFQTNLVPYPRIHFPLATYAPVISAEKAYHEQLSVAEITNACFEPANQMVKCDPRHGKYMACCLLYR
                    250       260       270       280       290       300       310       320
b-Tubulin  CLRFPGQLNADLRKLAVNMVPFPRLHFFMPGFAPLTSRGSQQYRALTVPELTQQMFDSKNMMAACDPRHGRYLTVAAIFR
            H7   T7      H8             S7     M loop         H9     H9′      H9"     S8

                     H10                    S9                     S10            H11
a-Tubulin  GDVVPKDVNAAIATIKTKRSIQFVDWCPTGFKVGINYQPPTVVPGGDLAKVQRAVCMLSNTTAIAEAWARLDHKFDLMYA
                    330       340       350       360       370       380       390       400
b-Tubulin  GRMSMKEVDEQMLNVQNKNSSYFVEWIPNNVKTAVCDIPP--------RGLKMSATFIGNSTAIQELFKRISEQFTAMFR
                     H10                    S9                     S10            H11

                 H11′             H12
a-Tubulin  KRAFVHWYVGEGMEEGEFSEAREDMAALEKDYEEVGVDSVEGEGEEEGEEY
                    410       420       430       440       450
b-Tubulin  RKAFLHWYTGEGMDEMEFTEAESNMNDLVSEYQQYQDATADEQGEFEEEEGEDEA
                 H11′             H12
```

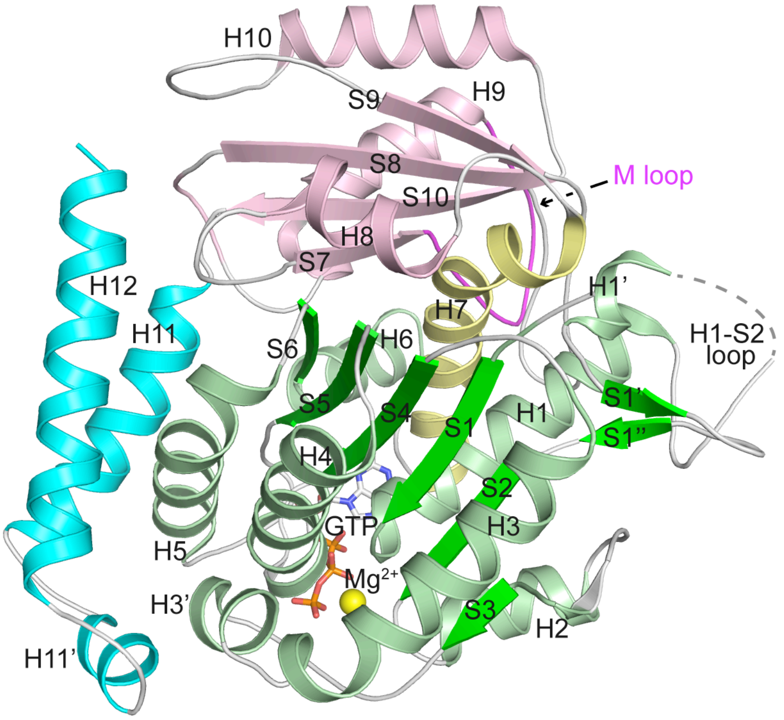

Figure S1. Sequences of α- and β-tubulin, secondary structure assignments and domain definition, Related to Figures 2, 3 and 5. (Top) The sequences of the *Bos taurus* α1B and β2B tubulin isotypes have been aligned as in (Löwe et al., 2001). These sequences were used to refine the tubulin–iE5 and tubulin–iiH5 structures because, to the best of our knowledge, those of *Ovis aries* tubulin are not known. The secondary

structure nomenclature is as in (Löwe et al., 2001) and the boundaries of the helices (highlighted in cyan) and strands (yellow) were determined with Pymol from the following structures: pdb id 6GWC (this work), 5EYP (Ahmad et al., 2016), and 4I4T (Prota et al., 2013a), which comprises a helical motif in the M loop as shown here. (Bottom) Tubulin domains. The α subunit (pdb id 5EYP) is shown. The secondary structure elements of its N-terminal domain are in green, with the strands in brighter color; those of the intermediate domain are in pink; and the helices of the C-terminal domain are in cyan. The H7 helix is in yellow and the M loop in magenta.

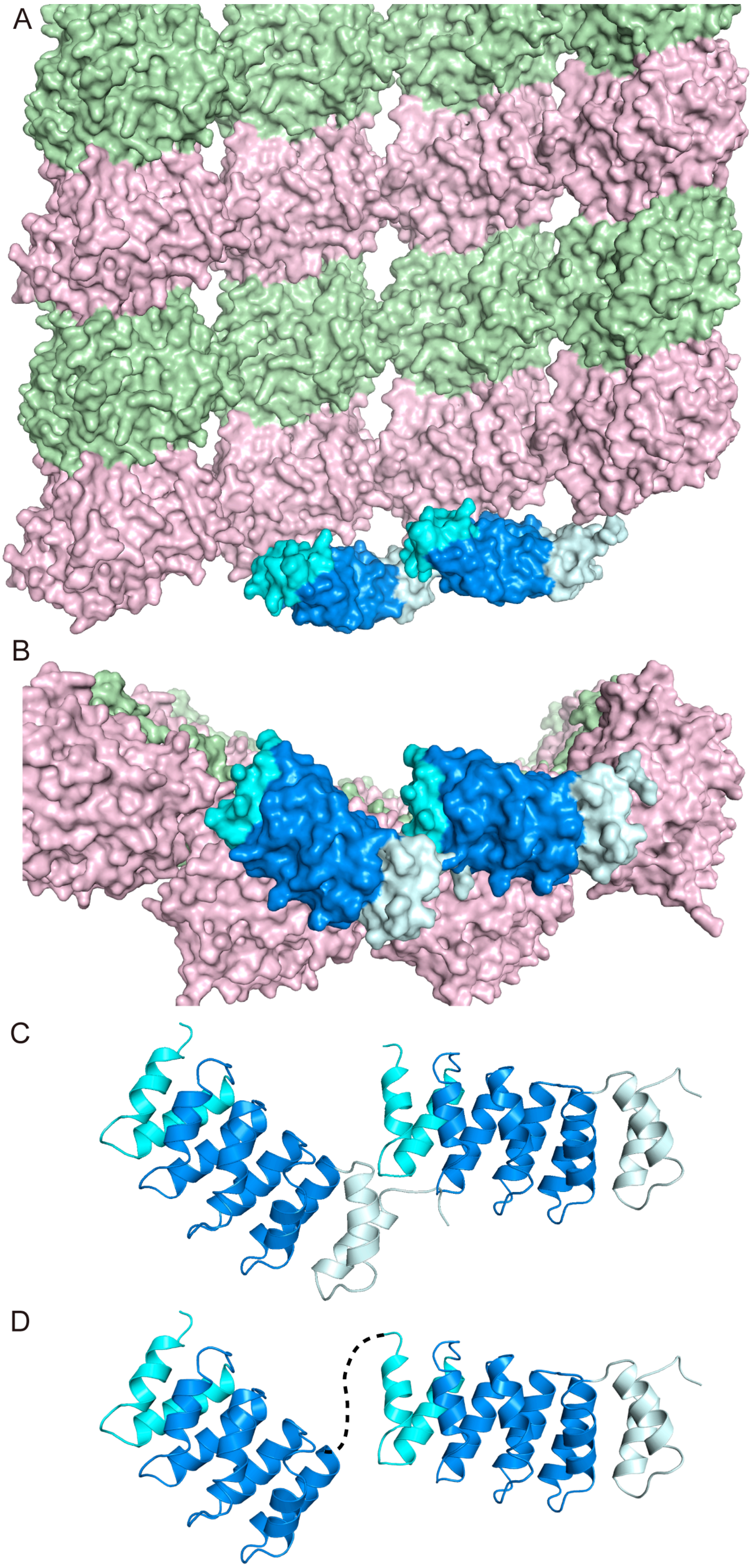

Figure S2. Design of the (iiH5)$_2$ tandem repeat αRep, Related to Figures 1H, 3 and 4. (A,B) Model of iiH5 bound at the (-) end of a microtubule. This model was obtained by superposing α-tubulin in tubulin–iiH5 to α-tubulin in the microtubule structure (pdb id 3JAK). Four protofilaments are drawn, two of them

being decorated by a iiH5 molecule. Views from the inside of the microtubule (A) and along the microtubule axis (B). Color code as in Fig. 3A except that the C-cap of iiH5 is in lighter cyan. (C) Same orientation as in panel B, but only the two iiH5 molecules are shown. (D) Design of $(iiH5)_2$. To build the tandem repeat αRep, the C-cap of one αRep was removed and the C-terminal end of its last internal HEAT repeat was linked to the N-terminal end of the N-cap of the second one using a $(GGGGS)_3$-GGS motif (dashed line).

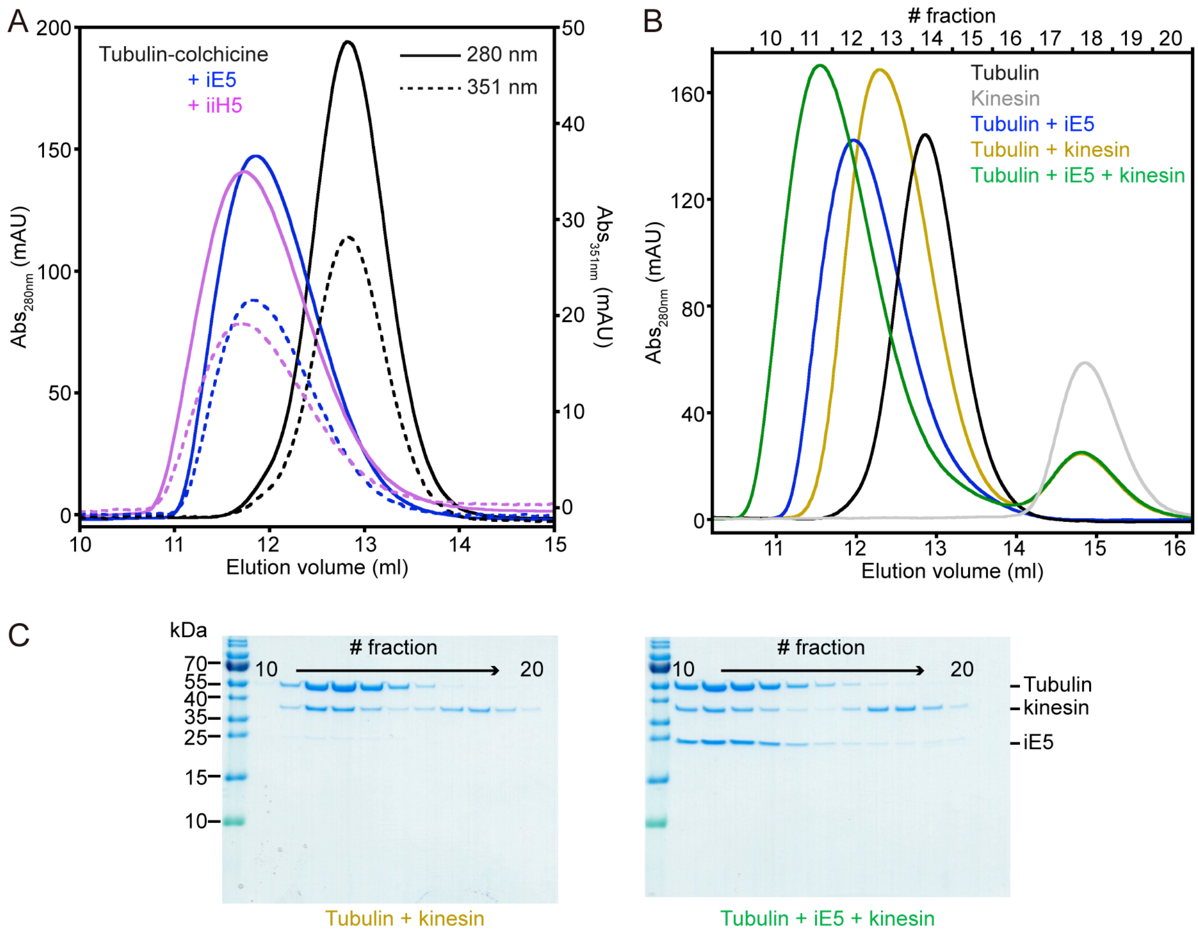

Figure S3. iE5 interacts with tubulin bound to colchicine or to kinesin-1, Related to Figures 1B and 5. (A) iE5 and iiH5 interact with the tubulin–colchicine complex. Gel filtration profile in 20 mM Pipes-K, pH 6.8, 1 mM $MgCl_2$, and 0.5 mM EGTA, of 20 μM tubulin–colchicine alone (black lines) or with 80 μM iE5 (blue) or iiH5 (magenta). The absorbance signal of colchicine at 351 nm is also shown (dashed lines). (B,C) iE5 makes a ternary complex with tubulin and kinesin-1. (B) Gel filtration profile in the buffer used in panel A of tubulin (20 μM), of kinesin-1 motor domain (30 μM), or of different mixtures of tubulin, kinesin, and iE5 (80 μM), as indicated. The main chromatographic peak of the tubulin:iE5:kinesin sample is shifted compared to those of tubulin:kinesin and tubulin:iE5, indicating the formation of the ternary complex. (C) Fractions defined at the top of panel B were submitted to SDS-PAGE in the case of tubulin:kinesin (Left) and tubulin:iE5:kinesin (Right), confirming the formation of a ternary complex in this last case.
